# The Association Between Affect and Adiposity in Childhood and Adolescence: A Systematic Review

**DOI:** 10.1111/obr.70056

**Published:** 2025-12-22

**Authors:** Emma S. Young, Alice R. Kininmonth, Yue Wang, Jason C. G. Halford, Clare Llewellyn, Alison Fildes

**Affiliations:** ^1^ School of Psychology, Faculty of Medicine and Health University of Leeds Leeds UK; ^2^ School of Food Science and Nutrition, Faculty of Environment University of Leeds Leeds UK; ^3^ Research Department of Behavioural Science, Institute of Epidemiology and Health Care University College London London UK

**Keywords:** adiposity, adolescence, affect, childhood

## Abstract

**Background:**

The relationship between adiposity and psychological health is complex, with much of the current research focused on adult or adolescent psychopathology. Affect, a facet of psychological well‐being observable from infancy, appears to influence energy balance behaviors, but relationships with adiposity are not fully understood. This review examined the association between affect and adiposity across childhood and adolescence to further understand the nature of these relationships.

**Methods:**

Six electronic databases were searched in February 2024. Studies that reported an association between measures of adiposity and affect were included and synthesized narratively. Study quality was assessed using an adapted version of the Newcastle–Ottawa Scale.

**Results:**

One hundred and sixty‐seven studies were retrieved from the search. Studies overwhelmingly focused on negative affect (*n* = 75) rather than positive affect (*n* = 14) or both (*n* = 21). Thirty‐three studies focused on adiposity and emotional functioning, and 24 on emotional regulation. Negative affect was more consistently associated with adiposity in adolescence. There was little evidence of bidirectionality, whereby higher adiposity generally preceded negative affect. Positive affect was also related to adiposity, although these relationships were mixed, with prospective associations found with higher and lower adiposity across development. Mechanisms of associations were infrequently examined but varied when reported.

**Conclusions:**

Positive and negative affect both appear to be associated with adiposity, and these relationships may be dynamic across development. Longitudinal research to elucidate these associations across development is necessary to confirm whether these trends are a true developmental phenomenon or a function of sample differences in baseline affect.

## 1 Introduction

Obesity is a complex, multifaceted disease, driven by the interplay of genetic, environmental, behavioral, and psychological factors [1]. In 2022, 390 million children and adolescents between the ages of 5 and 19 were overweight globally, of which 160 million were living with obesity [2]. Concurrently, the incidence of poor mental health among children and adolescents has risen over the past 30 years [3]. This dual increase has attracted growing interest in exploring whether and how the two are associated. Previous reviews have examined associations between adiposity and depression or anxiety, with a recent umbrella review finding “convincing” evidence for an association between depression, anxiety, and higher body weight in childhood and adolescence [4]. However, given that the first onset age for psychopathology is commonly around 14 years of age [5], the examination of affective disorders, such as depression, is less informative for identifying where preventative efforts against the development of both unhealthy weight and psychological problems may be best placed.

Elucidating relationships between adiposity and psychological health across development, particularly in younger children, requires going beyond psychopathology to examine related factors that may precede poor mental health. Previous reviews have also explored broader concepts of psychological wellbeing in this population, albeit adopting varied definitions that encompass diagnosed mental health conditions, flourishing, body dissatisfaction, or psychological safety [6, 7, 8]. The assumption that higher adiposity and poor psychological wellbeing are inevitably related has been both somewhat supported [7] and challenged [8], with these reviews highlighting methodological heterogeneity as a limiting factor. Studies tend to examine different facets of psychological wellbeing simultaneously or focus on facets that are not consistently salient across development. For example, children do not fully develop the ability to internalize criticism and engage in social self‐comparison until the age of 6 [9], suggesting explorations of body dissatisfaction as a proxy for wellbeing may not be informative for clarifying early relationships between adiposity and psychological health.

Given it is observable from infancy, affect, a superordinate term for emotions and moods [10], may be one factor that can be better and more consistently examined in relation to adiposity across development. Relationships between negative affect encompassing emotions such as sadness, anger, and nervousness, and obesity are hypothesized to be cyclically driven through over‐consumption in response to negative affect, and through body dissatisfaction resultant from excess weight, creating a theorized “circle of discontent” (CODT) [11]. Positive affect, including emotions such as excitement or happiness, has also been shown to influence eating behaviors in adults [12] and children [13, 14], with these relationships suggested to be independent from, rather than the inverse of, eating in response to negative affect [15]. However, no review has synthesized literature to explore relationships between both positive and negative affect with adiposity *outcomes* across the weight spectrum in childhood. Therefore, the current systematic review aimed to comprehensively examine the associations between affect and adiposity across childhood and adolescence, and the potential mechanisms of observed associations.

## 2 Methods

This review followed PRISMA guidelines [16] and was registered on PROPSERO (Registration number: CRD42024500525).

### 2.1 Eligibility Criteria

Studies were included if they were original, peer‐reviewed experimental, or observational research studies reporting on the association between a measure of adiposity, such as body mass index (BMI) z‐score, and a measure of affect.

Given high levels of negative affect can characterize common affective disorders, studies using global wellbeing or depression measures were also included if they reported on the association between subscale scores for an affective domain and an adiposity measure. Measures of temperament were also included in the review if they reported on dimensions of negative affectivity. Surgency, although a higher‐order factor that also includes approach behaviors, is a temperamental dimension frequently utilized in infant research [17] which is characterized by positive affect and pleasure and was therefore included in the review as a proxy measure of positive affect. Affect could be either self‐, teacher‐, or parent‐reported. Studies testing associations with exclusively physiological aspects of “core” affect (fatigue, feeling active, boredom, or liveliness) were excluded, unless they were assessed alongside valenced items of emotion or mood. The population of interest was children and adolescents ≤ 19 years, provided the sample mean age was < 18 years. Participants in receipt of pharmacological treatment for affective disorders were excluded, to avoid conflating the established side effect of medication‐related weight change [18] with changes in affect. Studies where the only measure of weight was birth weight were also excluded.

### 2.2 Database Search

Six electronic databases were searched from inception in February 2024: PsycInfo (OVID), Web of Science, PubMed, EMBASE (OVID), Medline (OVID), and EBSCO CINAHL. The full search strategy and search terms are listed in Files S1 and S2, respectively.

One reviewer (EY) screened all titles and abstracts, and a second reviewer (YW) reviewed a randomly selected sample of 5% (*n* = 1088). Ten percent of full texts were subsequently screened in duplicate (*n* = 189). The two reviewers agreed on ~95% of inclusion/exclusion decisions in both title/abstract and full‐text screening, and any disagreements were resolved through discussion. Authors were contacted where full texts were unavailable. An updated search for literature published between February 2024 and July 2025 was executed and titles and abstracts were reviewed to identify any recently published studies that might alter conclusions drawn from the original synthesis.

### 2.3 Data Extraction

A standardized data extraction form was created and piloted before the point of data extraction. Data pertaining to study characteristics (design, methodology) and sample (demographics); measures of affect and adiposity utilized; presence, strength, and directionality of relationships (e.g., beta coefficients, odds ratios), and mediators and moderators of any observed associations were extracted by one reviewer (EY). In all instances, where data from multiple statistical techniques or models were presented, data from the most adjusted model were extracted.

Where multiple reports arose from the same cohort, the study with the most comprehensive analysis was prioritized and retained to prevent double counting. Where two studies overlapped but each provided a unique finding on a different aspect of affect, both were retained but only the unique aspects were synthesized. Details of the selection process for multiple reports are outlined in File S3.

### 2.4 Study Quality Assessment

Quality of studies was assessed using the Newcastle–Ottawa Scale (NOS), adapted for the assessment of cross‐sectional studies [19]. The NOS was also used to assess experimental studies that tested associations through baseline data only, or that controlled for the intervention arm in their analysis, because the relevant analysis did not evaluate intervention effects. However, the possibility of residual confounding was assessed for these study types. Because of the volume of literature, one primary reviewer (EY) assessed the quality of all included papers, and a second reviewer (YW) independently cross‐checked a sample of 10% of these studies. Full details of study quality assessment are outlined in File S4.

## 3 Results

**FIGURE 1:**
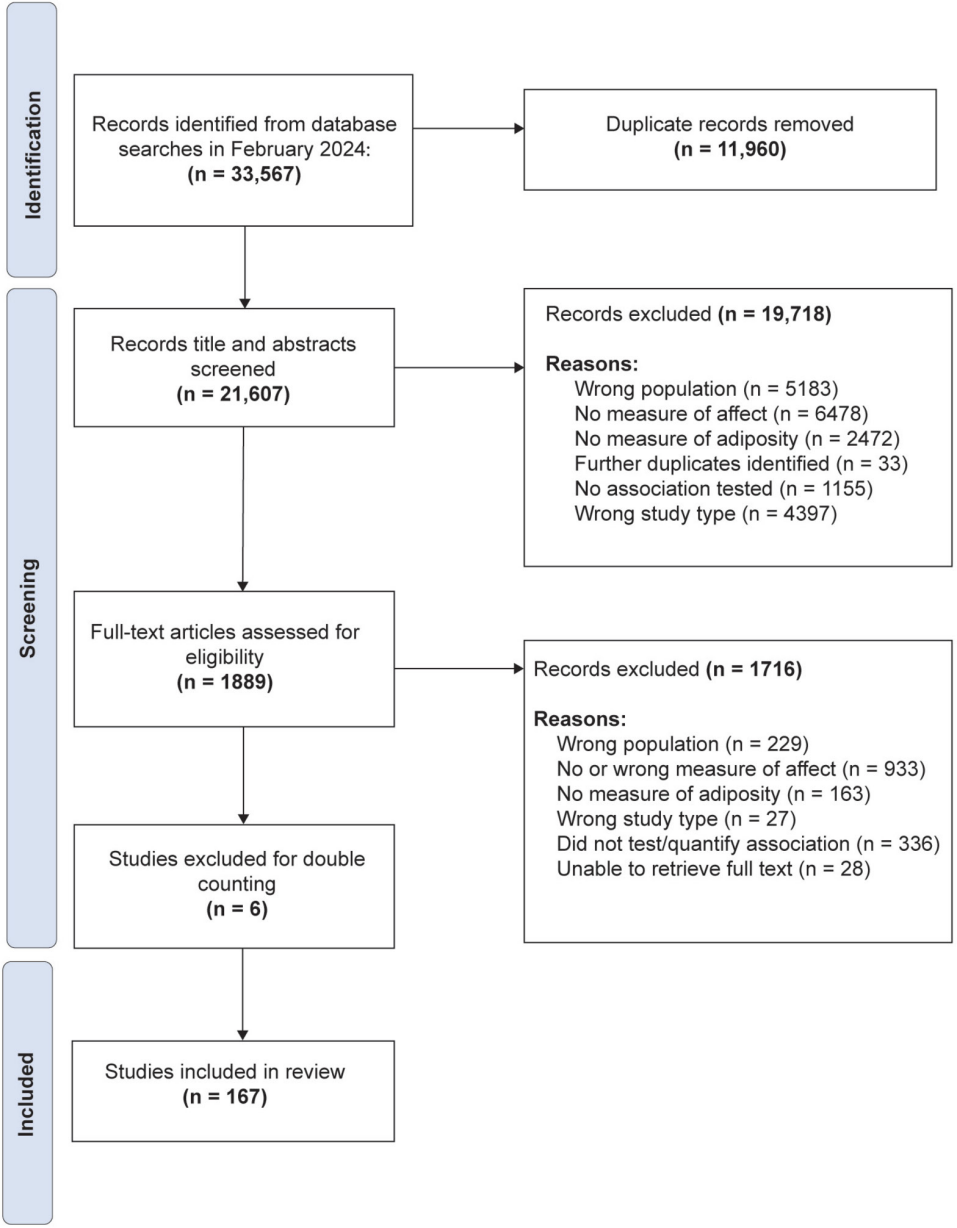
Flow chart of study selection process.

In total, 167 studies were included in the synthesis (Figure 1). Table 1 provides a brief overview of associations found between adiposity and affect, by study type and aspect of affect explored. Details of the included longitudinal studies (where baseline measures of the dependent variable were controlled for) are provided in Table S1 (*n* = 23) and nonlongitudinal studies (including prospective studies that did not control for baseline measures) in Table S2 (*n* = 105). To ensure emphasis is placed on articles of the highest statistical quality, articles that only quantified associations between affect and adiposity using simple correlations (*n* = 39) are summarized separately in File S5. Associations described hereafter only reflect the remaining 128 studies, detailed in Tables S1 and S2.

**TABLE 1:** Summary of associations found by study design and affect type.

Longitudinal studies (*n* = 23)			Headline findings
Adiposity → Affect			
*+ve Association*	−*ve Association*	*Null*	
PA = 0	PA = 0	PA = 0	• Evidence for an adiposity → affect pathway was mixed
NA = 4^a,b,c,d^	NA = 0	NA = 3^c,e^	• Where pathways were significant, ↑ adiposity at baseline predicted ↑ NA at follow up
EF = 0	EF = 0	EF = 0	• No studies examined relationships from adiposity to later PA or EF
ER = 1	ER = 0	ER = 0	
Affect → Adiposity			
*+ve Association*	−*ve Association*	*Null*	
PA = 0	PA = 1	PA = 1^a^	• Most studies found no evidence for a longitudinal NA → adiposity pathway
NA = 6^d^	NA = 1	NA = 9^a,b,c,e^	• Only ↑ NA at baseline longitudinally predicted ↑ adiposity at follow up
EF = 0	EF = 2	EF = 0	• ↑ EF at baseline predicted ↓ adiposity at follow up
ER = 0	ER = 1	ER = 2	
Prospective studies (*n* = 19)			
Adiposity → Affect			
*+ve Association*	−*ve Association*	*Null*	
PA = 0	PA = 1	PA = 4^f^	• Most studies showed no relationships between baseline adiposity and any type of later affect
NA = 2^f^	NA = 1^f^	NA = 2	• ↑ adiposity was only prospectively associated with later ↑NA
EF = 0	EF = 0	EF = 2	• Findings for adiposity → EF were null
ER = 0	ER = 1^g^	ER = 1^g^	
Affect → Adiposity			
*+ve Association*	−*ve Association*	*Null*	
PA = 3^h^	PA = 1^h^	PA = 1^i^	• ↑ PA at baseline was most consistently prospectively associated with later ↑ adiposity
NA = 1	NA = 0	NA = 2^i^	• Evidence for a NA → adiposity relationship was mixed
EF = 0	EF = 0	EF = 0	• No studies tested prospective associations between baseline EF and later adiposity
ER = 0	ER = 0	ER = 2	• Findings for ER → adiposity were null
Cross‐sectional studies (*n* = 86)			
*+ve Association*	−*ve Association*	*Null*	
PA = 0	PA = 7^k,m,n,o,p,q^	PA = 5^l,r,s^	• Associations were mixed for all types of affect
NA = 21^j,m,n,o^	NA = 2	NA = 27^j,k,l,p,q,r,s^	
EF = 3	EF = 7	EF = 9	• For NA, most relationships with adiposity were null, or showed ↑ NA associated with ↑ adiposity
ER = 1	ER = 5	ER = 5	• For PA, EF, and ER findings were mostly null or showed negative relationships such that ↑ adiposity was associated with ↓ PA/EF/ER.

*Note:* +ve = Positive; −ve = Negative; → = path direction; ↑ = increased; ↓ = decreased. Matching superscript letters across association groups denote mixed findings within the same study. Does not include simple correlation studies (*n* = 39). Temperamental and non‐temperamental affect combined for brevity.

Abbreviations: EF, emotional functioning; ER, emotional regulation; NA, negative affect; PA, positive affect.

### 3.1 Study Characteristics

Studies were published between 1985 and 2024 and derived from 29 countries, with the majority conducted in North America (*n* = 58) or Europe (*n* = 56). Samples were largely community‐based (*n* = 105), and only four studies tested associations with a pre–post design. Most examined associations with adiposity nonlongitudinally (*n* = 105), with fewer true longitudinal studies (*n* = 23). Across the studies, 31 tools were used to measure affect, the most common being the emotional functioning scale of the Pediatric Quality of Life Questionnaire [20] (PedsQL; *n* = 27), the emotional problems subscale of the Strengths and Difficulties Questionnaire [21] (SDQ; *n* = 21), and the State/Trait Anxiety Scale [22] (STAI; *n* = 13). Adiposity was typically measured through BMI (*n* = 112), which 42 studies used to categorize participants by weight status. A further 35 studies standardized BMI into z‐scores or standard deviation scores (SDS), and 18 into centiles. Six infant temperament studies used weight‐for‐length scores. Other measures included (used in conjunction with BMI): body fat percentages (*n* = 5), fat mass in kg (*n* = 2), waist circumference (*n* = 1), waist‐to‐hip ratio (*n* = 1), and waist‐to‐height ratio (*n* = 1). One study measured weight only (in pounds), and one infant study measured weight gain in grams.

Median follow‐up duration in longitudinal studies was 3 years, although this varied significantly (0.5–11 years). Sample sizes for community‐based studies varied significantly, ranging from < 100 participants to over 370,000; clinical studies (herein used to describe treatment‐seeking samples exclusively living with obesity) tended to have consistently smaller samples of ~100–200 participants, as did studies where the effect was observed in a laboratory setting (< 100–200 participants). Age ranges of samples were also heterogeneous but were particularly variable in clinical studies, which often recruited samples from across developmental stages, such as early childhood to later adolescence (e.g., 5–19 years [23], 2–18 years [24]).

Examinations of potential mediators and moderators were inconsistent and infrequent. Mediators included eating and feeding behaviors (*n* = 7), psychosocial factors (body image and esteem [*n* = 3]), weight teasing (*n* = 2), and parenting style (*n* = 4). Moderators (defined here as factors explored either through dedicated analysis of interaction terms or subgroup analyses) included eating and feeding behaviors (*n* = 4), sleep (*n* = 1), physical activity (*n* = 1), screen time (*n* = 1), and serum cortisol (*n* = 2). Most commonly, the moderating effect of gender was explored (*n* = 19).

Study quality (see File S4) was generally moderate to high, owing to consistent measurement of adiposity through clinician or researcher‐based methods (*n* = 109). As simple correlation studies were not screened for bias, all included studies also tended to score highly on the selection and justification of statistical methods (*n* = 110). Overall, the description of nonrespondents or loss to follow‐up was poor. While most studies controlled for age and gender, around one‐third failed to adequately control for important relevant confounders in the context of the study, such as not controlling for pubertal status in studies of pre‐ and postpubescent samples, or indicators of socioeconomic status in samples recruited across varied areas (lower income inner city vs. more affluent suburbs). Studies exploring group differences often did not match exposed and control groups for demographic factors.

### 3.2 Narrative Review of the Studies

Studies are reviewed narratively, because of heterogeneity in sample characteristics and measurement tools used. Studies of temperamental affectivity (*n* = 23) are discussed independently from non‐temperamental affectivity. This is to account for necessary differences in how affect is defined and assessed in infancy (i.e., surgency, where emotional expression is assessed alongside approach and sociability behaviors as a proxy for positive affect). While all concepts have overlap, studies of emotional functioning and regulation are discussed separately for clarity, as they reflect behavioral and cognitive expressions of affect, rather than the experience of affect alone. Emotional functioning is primarily focused on the child's experience of issues related to, and the behavioral expression of affect, while emotional regulation focuses on the child's ability to recognize, understand, and manage emotions, and their ability to utilize the necessary cognitive skills to do so.

#### 3.2.1 Temperamental Negative and Positive Affect

The review identified 23 studies of temperament, with 14 studies focused on negative affect [25, 26, 27, 28, 29, 30, 31, 32, 33, 34, 35, 36, 37, 38], four on positive affect [39, 40, 41, 42], and five on both positive and negative affect [43, 44, 45]. All temperament studies (longitudinal *n* = 9/23, nonlongitudinal *n* = 14/23) were conducted with children aged < 11 years. They were largely community‐based (22/23), with only one study conducted in a clinical setting [29]. Assessment tools varied, but temperament was most commonly measured with the Child Behavior Questionnaire (9/23) [46]. Three of the studies measured temperament through task batteries and researcher observation [31, 41, 42].

Negative Affectivity. Four (4/7) of the longitudinal studies examining negative affectivity found no direct association with adiposity [28, 29, 36, 38]. Three longitudinal studies (3/7) did report direct associations between negative affectivity and adiposity. These samples varied in age from infancy to mid‐childhood and yielded disparate findings. A study of infants in the United States (*n* = 200) found weight‐for‐length gain between 6 and 12 months of age was associated with more frequent crying and fussing [26]. However, a second UK study (*n* = 62) found negative affectivity at 3–5 years was associated with a lower BMI z‐score 2 years later [27]. A third US study reported negative affectivity was associated with higher instability in growth trajectory between 4.5 and 9 years [33].

Two of the seven nonlongitudinal studies of negative affectivity found direct associations with adiposity in children under 3 years of age [31, 37]. Again, findings were mixed—Kong et al. [31] found negative affectivity during play was positively associated with weight‐for‐length z‐score in US infants aged 9–15 months (*n* = 121), while Stifter et al. [37] found negative affectivity was negatively associated with BMI‐for‐age z‐score in infants and children aged 3–36 months from the United States (*n* = 100). Liew et al. [32] did not find direct effects but observed a positive indirect effect of negative affectivity on weight status in children aged 4–6 years (*n* = 221), through food approach and restrictive feeding. The remaining studies (4/7), all conducted with children aged between 3 and 8 years, found null associations [25, 30, 34, 35].

Positive Affectivity. Four studies focused exclusively on positive affect. The only longitudinal study found that positive affect during play at 1 month of age was associated with a normative growth trajectory between 1 month and 6 years in children from the United States (*n* = 216) [41]. Three further prospective studies from the United States found positive relationships between positive affect and adiposity. The first found that surgency at age 5 was associated with a higher BMI z‐score in early adolescence (*n* = 180) [40]. A second found that higher reported levels of laughing and smiling at age 2 were associated with a larger increase in BMI z‐score between the ages of 4 and 10 (*n* = 195) [39]. The third study found that positive affect at 4 months old was associated with a higher BMI z‐score and body fat percentage at 12 months, but not cross‐sectionally (*n* = 126), and relationships varied by the context in which positive affect was observed (social vs. nonsocial) [42].

Mixed Affectivity. Five studies assessed relationships between both positive and negative affectivity and adiposity, of which one was longitudinal. Four (4/5) studies reported some associations, but none found direct relationships between both positive and negative affect and adiposity simultaneously. A study of UK infants (*n* = 75) found that slower weight gain between birth and 8 weeks of age was associated with greater observed fear and negative emotionality, but not smiling and laughter, at 8 weeks [43]. Between the ages of 2 and 6 years, surgency was negatively associated with BMI z‐score in US children (*n* = 100), although indirect effects for negative affectivity and higher BMI z‐score were found through authoritarian parenting style [47]. Leung et al. [48] found an indirect relationship between adiposity and positive affect, such that surgency was positively associated with concurrent BMI z‐score through enjoyment of food and food responsiveness in 4‐year‐olds from the United States (*n* = 379). However, no effects were observed for BMI z‐score change and no association was observed for negative emotional lability. A larger study of 4‐year‐old children from the United States (*n* = 718) found no association between surgency or negative affectivity and BMI z‐score [44]. The single longitudinal study that examined both surgency and negative affect at 6 months, found no association between either kind of affect and an increase in weight‐for‐length z‐score between 6 and 12 months in US children (*n* = 216) [45].

#### 3.2.2 Non‐Temperamental Negative and Positive Affect

The review identified 61 studies of non‐temperamental negative affect and positive affect (longitudinal *n* = 8/61, nonlongitudinal *n* = 53/61) conducted with children aged 3–19 years of age. The majority were conducted in community samples (51/61). All longitudinal studies, and 8/10 of the clinical studies examined negative affect only.

Negative Affect. Forty‐four studies (36/44 = nonlongitudinal) examined associations between negative affect and adiposity, outside of a temperament context. Emotional problems, measured by the SDQ [21] (*n* = 21/44) and anxiety symptoms (15/44), primarily measured through the State/Trait Anxiety scale [22] (*n* = 13/44) were the most commonly explored “types” of negative affect. All eight longitudinal studies were conducted with samples recruited from the community.

Five out of eight longitudinal studies assessed bidirectional associations, all using the SDQ [49, 50, 51, 52, 53]. One study (1/5) also used a summed score of three single items for anxiety, anger, and sadness (“negative emotions”) [52]. One study (1/5) found null associations in either direction [50]; three studies (3/5) found evidence for a relationship from adiposity to negative affect, but that negative affect did not predict adiposity [49, 51, 52]. Creese et al. reported a unidirectional association between BMI z‐score at 11 years and emotional symptoms at 17 years in UK boys, but no direct association for girls (*n* = 12,450). However, indirect associations between BMI z‐score at age 11 and emotional symptoms at age 17 were found through happiness with appearance and self‐esteem (measured at 14 years) for both boys and girls. For boys only, emotional symptoms at age 11 also indirectly predicted BMI z‐score at 17, through happiness with appearance, but not self‐esteem at age 14. Patalay and Hardman [53] found lagged bidirectional associations dependent on age in children from the same UK cohort (*n* = 17,215), such that BMI at age 7 predicted emotional symptoms at age 11, but not the reverse; similarly, emotional symptoms at age 11 predicted BMI at age 14, again with no evidence for the reverse once socioeconomic factors were controlled for. No relationships were observed in younger children (3–7 years). Michels et al. [52] found adiposity at age 6–11 years was associated with greater negative emotions at age 7–12 years in Belgian children (*n* = 316), but negative emotions at age 6–11 years did not predict adiposity 1 year later. Null associations were observed at other ages. A cross‐sectional study of the same cohort again found no association between BMI z‐score or body fat percentages and the SDQ, or any other negative emotions at ages 5–10; however, the study did find that happiness was inversely associated with adiposity in this period [54].

Cross‐lagged associations in Michels et al.'s study [52] were also only observed for the summed single items for negative emotions (anger, anxiety, sadness), not for the SDQ score. Another longitudinal study of US adolescents (*n* = 213) examined multiple discrete affective states in isolation rather than summed together, and found anger, but not anxiety, was associated with adiposity 3 years later [55]. The remaining two longitudinal studies reported no direct associations between age 3 and 5 years [56] and 7 and 11 years [57]; however, the latter study did find indirect effects of higher BMI at age 7 and greater emotional problems at age 11, through weight bias internalization and weight‐based teasing (*n* = 1047).

In all but one clinical study of negative affect (7/8), positive associations between adiposity and negative affect were found, such that higher adiposity was associated with more emotional problems [58, 59], anxiety [60, 61, 62, 63], and anger [64]. This was generally explored through group differences, dichotomizing samples into those living with obesity, and those of “normal” weight (5/8). Two studies measured BMI z‐score alongside weight status (*n* = 2) [60, 64] one of which found a null association between BMI z‐score and trait anxiety despite significant differences by weight status [64]. A further four community studies of anxiety found positive associations with adiposity [65, 66, 67, 68], and five showed null associations [69, 70, 71, 72, 73]. In the remaining community studies of negative affect, seven reported a direct positive association with adiposity (7/21) [74, 75, 76, 77, 78, 79, 80] and one found a negative association [81]. One Norwegian study (*n* = 1088) observed this relationship to be curvilinear, such that children who were underweight or living with obesity experienced more emotional problems than normal weight or overweight children [74]. Five studies found associations were moderated by either age (2/5) [51, 79], gender (3/5) [78, 80, 81], and levels of physical activity (1/5) [80]. A study of Australian children (*n* = 3197) found associations between emotional symptoms and adiposity only emerged at 10–11 years of age, with null or weak associations beforehand [49]. Förster et al. [79] (*n* = 2350) only observed cross‐sectional associations between negative affect and adiposity in German children and adolescents aged 11–18 years, and not in those aged 4–10 years. In adolescent samples, Ivarsson et al. [81] (*n* = 405) and Bjertnaes et al. [78] (*n* = 3189) both found associations between adiposity and negative mood for girls, not boys, although these associations were negative and positive respectively. Noonan and Fairclough [80] found that negative affect and BMI were positively associated only in boys in the second least‐active quartile of their sample of 7‐year‐old UK children (*n* = 6011).

The remaining 10/36 nonlongitudinal studies reported null associations [82, 83, 84, 85, 86, 87, 88, 89, 90, 91]. Three of these studies did, however, find indirect associations, such that negative affect was associated with loss‐of‐control eating in 8‐ to 17‐year‐olds (*n* = 257) [71], with uncontrolled eating, restrictive eating, and body dissatisfaction in young adolescents (*n* = 282) [92], and with emotional eating and food responsiveness in 3‐ to 4‐year‐olds (*n* = 194) [93].

Positive Affect. Six studies investigating associations between adiposity and positive affect were identified [94, 95, 96, 97, 98, 99]. All studies operationalized this as happiness (6/6), and each study used a different measurement tool. Three studies were prospective (3/6), but none were longitudinal, and all were conducted with community‐based samples.

Two studies found negative associations (2/6). A prospective study of UK children (*n* = 16,936), found a “high increasing” or “moderate increasing” BMI trajectory between the ages of 3 and 11 that was associated with lower happiness scores at age 11 [95]. A second cross‐sectional study of South Korean adolescents grouped by weight status found that adolescents categorized as underweight were happier than those of a “normal” weight [96]. The remaining four studies (4/6) found null associations [94, 97, 98, 99].

Mixed Valence Affect. Ten studies (*n* = 1 clinical study) assessed associations between adiposity and affect using mixed valence measures. None were truly longitudinal; however, two were prospective. Seven of the 10 studies reported relationships between adiposity and affect. Five of these studies (5/7) reported that lower positive affect was associated with higher adiposity, but only three (3/5) also observed positive relationships between negative affect and adiposity [100, 101]. One study (1/7) was unable to determine whether low positive or high negative affect was driving associations with adiposity because of the scoring method [102]. The remaining three studies of mixed affectual valence (3/10) found no associations between either kind of affect and adiposity [103, 104, 105].

Half (5/10) of the studies assessed mixed valence affect with the Positive and Negative Affect Schedule (PANAS) [106], although version use and scoring protocols varied. Two of the studies employing PANAS (2/5) used a total score, summing negative affect and the inverse of the positive affect scales together [102, 105]. In the first study, higher combined PANAS scores were found cross‐sectionally in children with an “intermediate” degree of obesity and those with a “mild” degree of obesity in a clinical sample of Chinese children and adolescents aged 4–15 years (*n* = 72) [102]. The second, a prospective community study of 208 UK children, found no association between changes in body fat percentage between age 7 and 16 years, and combined PANAS scores at age 16 [105]. Two of the remaining PANAS studies (2/5) found that in a sample of 412 Australian children aged 8–11 years [101], and a sample of 786 Spanish children aged 10–13 years [107], those living with overweight or obesity scored lower in positive affect than their non‐overweight counterparts. The former study also observed higher negative affect scores for the children with overweight than those without, while the latter study found no group differences for negative affect. In the latter study, affect was assessed in the context of physical activity, and the group differences for positive affect were largely driven by items of a physiological nature (feeling energetic and active) rather than those more closely associated with mood or emotion.

Two studies used single items to assess discrete positive (i.e., happiness) and negative (e.g., sadness, nervousness, anger) affective states (2/10) [54, 108] and two used a visual analogue scale (VAS) (2/10) [104, 109]. The two VAS studies were both conducted in Brazil and yielded mixed results: overweight or obesity were associated with a higher prevalence of “unfavorable” self‐reported emoticon scale scores in 6‐ to 10‐year‐olds (i.e., the 3 least “happy” emoticons) (*n* = 1048) [109]; while no associations were found in a sample of 11‐year‐old children (*n* = 4426) [104]. The two studies using single items to assess discrete affective states both found happiness was negatively associated with adiposity [54, 108]. However, one found that anxiety, anger, and sadness were not associated with adiposity [54], while the other found nervousness, and a “bad temper” were positively associated with adiposity [108].

#### 3.2.3 Emotional Functioning

Twenty‐seven studies assessed associations between adiposity and emotional functioning, of which the majority were nonlongitudinal (*n* = 25/27). Ten studies, including two longitudinal, were conducted in a clinical setting (10/27) [23, 24, 110, 111, 112, 113, 114, 115, 116, 117], and 17 were community‐based (17/27) [118, 119, 120, 121, 122, 123, 124, 125, 126, 127, 128, 129, 130, 131, 132, 133, 134]. All 27 studies utilized the emotional functioning subscale of the PedsQL.

Overall, 15/27 studies reported an association, eight from a clinical setting (8/10), while only 7/17 community studies reported any association. Where associations were observed, these were largely negative (*n* = 14), whereby higher adiposity was associated with worse functioning. One community study of Fijian adolescents (*n* = 8947) reported a negative association between weight status and emotional functioning among 15‐ to 18‐year‐olds, but a positive association in 12‐ to 14‐year‐olds. A further two studies similarly found age‐dependent associations; one UK clinical study of 5‐ to 16‐year‐olds (*n* = 540) found prepubescent children living with obesity reported the lowest functioning, while a Fijian community study reported positive cross‐sectional associations between functioning and BMI‐z score in boys, but not girls at age 15 and the same association in girls but not boys at age 17. Three studies found associations between adiposity and emotional functioning were contingent on who was reporting the PedsQL. In two cases, the association between functioning and adiposity in children < 12 years was only found in parent‐reported, not self‐reported functioning, in both community and clinical settings. The remaining 12 studies found null associations [114, 120, 121, 122, 123, 125, 126, 127, 129, 131, 133, 134].

#### 3.2.4 Emotional Regulation

Eighteen studies (4/18 longitudinal) explored associations between adiposity and emotional regulation, and 10/18 were conducted in community samples and 8/18 clinical samples. Emotional regulation measurement tools were diverse (*n* = 8) and scoring conventions were inconsistent, such that higher scores sometimes reflected better or poorer emotional regulation depending on the measure. The Difficulties in Emotion Regulation Scale (DERS) [135] was the most used measure (4/18). Nine studies reported that greater adiposity was associated with poorer emotional regulation skills [136, 137, 138, 139, 140, 141, 142, 143].

Two of the four longitudinal studies found conflicting associations. In younger children from the United States (aged 2 years), better emotional regulation was associated with a lower likelihood of BMI z‐score change 3.5 years later (*n* = 57) [137], while in US adolescents (*n* = 79), higher BMI at age 15 was associated with better emotional regulation 1 year later [144].

Five clinical studies found associations (5/8). In three of these, emotional regulation was measured with the DERS scale in Turkish adolescents grouped by weight status, all of which found that children living with obesity reported poorer emotional regulation [139, 140, 141]. In a sample of Italian children aged 6–12 years (*n* = 200), poorer emotional regulation, but not negative emotionality, was associated with obesity [143]. While an experimental study of German adolescents (*n* = 153) found change in BMI from pre‐ to postobesity treatment course was predicted by higher levels of emotional reappraisal, but not emotional suppression (i.e., better emotional regulation skills) at baseline [142].

Three studies found indirect associations between emotional regulation and adiposity. A prospective, community study of 2587 children in the United States found that poorer emotional regulation at age 5, measured through the Child Behavior Checklist, was associated with overeating at age 9, which in turn was associated with BMI z‐score at age 15 [145]. A second, smaller (*n* = 156) US‐based study found that greater emotional regulation at age 15 was associated indirectly with lower adiposity at age 19, through decreased emotional eating, but not increased dietary restraint at age 16 [146]. A third clinical study of adolescents aged 11–18 years in Türkiye (*n* = 123) found an indirect association between poorer emotional regulation and higher BMI SDS, through uncontrolled eating and internet addiction [147]. The remaining six studies reported null associations [148, 149, 150, 151, 152, 153].

#### 3.2.5 Moderating Factors and Effects

In total, 32 studies explored moderators of affect–adiposity associations. Most commonly included moderators were gender (*n* = 19) and age (*n* = 9).

Evidence for a moderating effect of gender was mixed, with most studies reporting no impact of gender on adiposity–affect relationships, in particular for emotional functioning (5/6 nonsignificant) [113, 115, 116, 123, 126]. Some studies did observe gender differences in the strength and significance of the adiposity–affect relationship, namely differences in negative [49, 78, 80, 81] and mixed valence affect [98, 105]. However, across the studies, no consistent pattern emerged to suggest stronger relationships between adiposity and any type of affect for either boys or girls.

Differences in affect–adiposity relationships by age appeared to be slightly clearer, particularly for negative affect. Longitudinal positive associations between negative affect and adiposity appeared to emerge from late childhood into preadolescence [49, 50, 52, 53]. Cross‐sectional subgroup analyses of discrete age categories also appeared to support this [79]. Emotional functioning studies were fewer, but findings indicated greater adiposity was associated with either increased or decreased emotional functioning depending on the age of the subsample [116, 130].

Exploration of other possible moderating factors (such as sleep, loss‐of‐control eating, or serum cortisol) yielded heterogeneous results. These moderators were infrequently reported across all affect types (often only appearing in one study).

### 3.3 Supplementary Searches

An updated search was conducted in July 2025 and abstracts were screened, to identify whether any studies published subsequent to the original search (February 2024) changed conclusions derived from the original search. Ten studies that satisfied inclusion criteria were found, seven of which were focused on negative affect (1/7 examining temperamental negative affect) [154, 155, 156, 157, 158, 159, 160], and 3/10 focused on emotional regulation [161, 162, 163]. The findings of these 10 studies were in line with the main synthesis and did not alter conclusions.

## 4 Discussion

This review examined literature exploring associations between adiposity and affect, including temperamental affectivity, across development. Included studies, predominantly focused on negative affect (*n* = 53) as opposed to positive affect (*n* = 10) or positive and negative affect concurrently (*n* = 15). Findings were mixed, with the most consistent associations reported in clinical samples, particularly between higher adiposity and poorer emotional functioning and regulation. Limited evidence was found for bidirectional associations between affect and adiposity, with more studies reporting adiposity predicting later negative affect rather than the reverse. The few studies that explored positive affect showed mixed relationships, with positive affect prospectively associated with both higher and lower adiposity across childhood and into adolescence.

The strength of associations between adiposity and affect in clinical samples is consistent with previous reviews of other wellbeing‐related concepts [8]. This might be, in part, because problems with emotional regulation and functioning are more easily observed as behavioral expressions of affect and therefore might elicit treatment‐seeking for childhood obesity more readily.

This review found limited evidence for bidirectional associations between affect and adiposity. Three of the longitudinal studies testing bidirectionality between affect and adiposity found adiposity preceded emotional problems, rather than the reverse [49, 51, 52], and studies examining longitudinal changes from early childhood to adolescence found null, or weak, associations in either direction preceding the middle‐childhood period. There was some suggestion these relationships may vary by age, with one study of 3‐ to 11‐year‐olds (*n* = 17,215) [53] reporting BMI as early as age seven predicted emotional problems at age 11, and emotional problems at age 11 predicted BMI at age 14. Several nonlongitudinal studies found negative affect was associated with either higher or lower adiposity in adolescents [76, 78, 79, 81], while studies conducted in middle childhood, preadolescence [79, 83, 84, 85], and younger children [29, 30, 34, 35, 36, 48, 82] mostly yielded null associations. However, in infancy, negative affect and early weight gain appeared more strongly correlated [26, 31, 37, 43], consistent with previous systematic reviews indicating that temperamental associations with weight outcomes were strongest in infancy, rather than in early childhood [164].

Conversely, the most compelling evidence for an association between positive affect and adiposity was derived from studies of early–middle childhood [39, 40, 48], with some limited evidence also observed in infants and toddlers [42]. Studies demonstrating significant relationships between positive affect and adiposity (*n* = 7) tended to be prospective, with substantial follow‐up periods (e.g., 7–8 years), and focused on temperamental, or trait aspects of affect. These studies demonstrated that early temperamental surgency [40] or a tendency to smile or laugh in infancy [39] predicted both higher adolescent BMI [40] and greater BMI change between ages 4 and 10, respectively [39]. However, when adiposity was tested as a predictor of later positive affect, the reverse relationship was observed, whereby a trajectory of increasing BMI through early and middle childhood predicted reduced odds of happiness in preadolescence [95]. Cross‐sectional studies, or those with shorter follow‐up periods (1–2 years), generally found positive affect to be unrelated to adiposity (4/6). However, where significant associations were observed (2/6), higher positive affect was associated with lower adiposity, both in adolescence [96], and from early childhood to preadolescence [95]. Only one study (*n* = 216) in this review used a truly longitudinal design, including a baseline measure of the examined outcome. This study found that positive affectual temperament at 1 month predicted a *normative* trajectory of weight gain up to age 6 [41], suggesting prospective relationships observed in other studies might instead be a function of higher baseline adiposity. However, it is difficult to draw conclusions based on a singular study, and no longitudinal studies measured positive affect at multiple time points across development.

Findings from the few studies that examined mixed valence affect were again varied. Three of 10 studies found adiposity was associated with more negative and less positive affect simultaneously in middle childhood and adolescent samples [100, 101, 108]. Two further studies, conducted in adolescents and early–middle childhood, observed associations between higher positive affect and lower adiposity, but no association between adiposity and negative affect [54, 107]. Taken together, the findings of the review suggest that associations between affect and adiposity may be dynamic over the course of childhood and adolescence, influenced by affectual valence and developmental stage.

The CODT does not outline a temporal sequence in which negative affect and adiposity influence one another. It is possible that the cyclical association between negative affect and adiposity may unfold over years, potentially explaining why bidirectional effects observed by Patalay and Hardman [53] appear to be lagged over the course of 7 years. The longitudinal studies in this review also suggest higher adiposity in middle childhood to preadolescence, a “critical” period in development for shaping body image [165], precedes negative affect. This suggests the psychosocial consequences of higher adiposity may be the “starting point” of any such cycle emerging [49, 51, 52].

The main mechanism proposed by the CODT, between affect and subsequent adiposity, is the consumption of energy‐dense foods (i.e., emotional overeating) to downregulate negative affect, which can be reversed through improved affect and subsequent weight loss [11]. However, the longitudinal studies in this review provide little evidence to support these relationships in childhood and adolescence, and systematic reviews in adults suggest higher food and caloric intake may be linked more strongly to positive affect [12, 166]. Although the main focus of this review was to examine associations between affect and adiposity outcomes, some limited evidence suggests both positive and negative affect could influence adiposity in early childhood through unique and even shared appetitive pathways. Food approach behaviors were associated with surgency and BMI z‐score in children aged 4–6 years [48], while also associated with negative affect and adiposity in other samples of children of a similar age [93], and nationality [32]. Elsewhere, a lab‐based study of 125 young adults and children aged 9–10 years observed, when free from parental influence, that school‐aged children reported a desire for the consumption of unhealthy snacks in response to positive affect more so than negative affect, while young adults were more likely to show the same desire in response to negative affect [13]. Given this review found evidence for a relationship between positive affect and higher adiposity in younger children, it is possible that positive affect and adiposity are indirectly associated in this early period through appetitive behaviors influencing adiposity development until middle childhood to preadolescence. It is then possible that the relationship inverts and adiposity becomes more strongly associated with negative affect through the psychosocial consequences of higher adiposity during the “critical period” of early adolescence. However, only one study attempted to directly verify the CODT, with indirect associations observed between adiposity and negative affect through body dissatisfaction and both uncontrolled and restricted eating in adolescents [92] (*n* = 282). However, the study's cross‐sectional design prevents the temporal precedence of these factors from being discerned.

No study in the review attempted to verify the CODT model preceding adolescence, nor did any study longitudinally explore the influence of both positive and negative affect on adiposity within the same sample. Therefore, it cannot be ascertained whether the differing influence of affectual states on appetitive traits and subsequent adiposity across developmental periods, seen in the studies across the current review, is simply a function of sample differences in baseline affect. Longitudinal models could better elucidate the intraindividual dynamics of the associations between both positive and negative affect with adiposity, and how these relationships might unfold over the course of development. Furthermore, while eating in response to negative affect is thought to be a learned, rather than inherited, behavior [167], the studies in this review suggest other, more heritable food approach behaviors, such as food responsiveness and enjoyment of food [168], may be associated with adiposity and driven by positive affect. Future intervention efforts could benefit from a clearer understanding of how etiologically different appetitive traits are associated with the affect–adiposity relationship across childhood.

This review also highlights the possibility of gender differences in relationships between adiposity and affect. Contrary to findings of previous reviews focusing on depression, where associations between depression and obesity were proposed to be more apparent in females [169], relationships with affect seem more complex. Unique relationships between affect and adiposity were reported for both boys and girls in five studies but the mixed findings and relatively few studies reporting differences by gender make it difficult to draw firm conclusions.

### 4.1 Strengths and Limitations of the Review

Unlike previous reviews which have targeted varied and age‐dependent aspects of psychological wellbeing [6, 169], this review focused on affect, a construct observable throughout development. Despite this, study designs and measurement tools were heterogeneous. The exception was for emotional functioning, where all included studies utilized the PedsQL. Affect assessment tools often contained items pertaining to constructs correlated with, but not solely representing affect, such as somatic symptoms (e.g., experiences of headaches), or approachability in infants. While indicative of the interconnectedness of affect with social, physiological, and cognitive factors in real‐world functioning, the inability to disentangle their influence limits firm conclusions about affect–adiposity relationships. The conceptualization of positive affect and mixed affect was also inconsistent across studies. While almost all studies operationalized positive affect as happiness, some psychometric measures equated low happiness scores (i.e., not being happy) with unhappiness. These are, arguably, two distinct affective states that should not be conflated; not being happy, rather than necessarily implying sadness or unhappiness, instead may reflect frustration, anger, or anxiety, or perhaps affectual states with no obvious valence attached (e.g., boredom). This review also highlights how discrete, but similarly valenced affectual states may have unique relationships with adiposity, with fear, anger, and sadness all found to influence weight differentially [54, 55].

The majority of studies used affect measurement tools that are widely utilized across different cultures. However, differences in cultural expectations about childhood behavior and norms related to emotional expression and socialization processes may influence how parents and teachers perceive and report children's behavioral expressions of affect. Given the majority of studies were conducted in Europe or North America, the cross‐cultural generalizability of review findings must be considered with caution. Studies frequently included samples with large age ranges, often with significant crossover between developmental periods, despite several studies observing age‐dependent associations [51, 52, 53, 79, 132]. The large age span of samples within included studies makes it difficult to identify potential mechanisms underlying associations, which may be more salient at different points in development (e.g., parental feeding practices in infancy vs. body dissatisfaction in adolescence). Additionally, relatively few of these studies controlled for important confounders, potentially obscuring the true nature of these associations further. Particularly for samples with age ranges into adolescence, while age and gender were generally well controlled for, pubertal status was not, despite the fact that puberty is associated with both emotional development and rapid anthropometric changes independent of age.

Finally, few studies examined relationships between affect and underweight, with children classified as underweight often grouped with “normal” weight children or excluded altogether. This review did identify some limited evidence that underweight children report being happier compared with normal weight children [96], or (along with those living with obesity) experience more negative affect than those who are overweight or normal weight [74]. Nonlinear relationships between adiposity and facets of psychological wellbeing are evidenced elsewhere, including subjective wellbeing in adults [170, 171], depression in adolescence [172], and emotional problems in children [173]. Including children and adolescents who are underweight could further understanding of how adiposity and affect are related across the weight spectrum and better characterize the linear properties of the relationship.

## 5 Conclusions

This review suggests relationships between affect and adiposity may be dynamic across development, possibly driven by differences in affectual valence, and underpinned by diverse mechanisms that are most salient at different developmental stages. The studies reviewed indicate that adiposity appeared to more frequently precede negative affect, rather than the reverse; the possible role of early positive affect in influencing adiposity over substantial periods of time was also evidenced. However, the limited number of longitudinal, bidirectional studies—as well as the considerable heterogeneity in the conceptualization and measurement of affect—hinder the ability to draw definitive conclusions. Nonetheless, this review provides a comprehensive examination of published literature pertaining to the adiposity–affect relationship to date. Further clarity regarding the direction and strength of these relationships across different stages of childhood and across cultures is needed. The prospect of links between poor psychological health and obesity presents opportunities for addressing two major public health concerns simultaneously. However, greater understanding of how these complex relationships develop, and how they might differ between children, adolescents and adults, is imperative for informing robust obesity policy and early intervention to promote emotional wellbeing for children across the weight spectrum.

## Conflicts of Interest

The authors declare no conflicts of interest.

## Supporting information


**Data S1:** Supporting Information

## Data Availability

The data that supports the findings of this study are available in the supplementary material of this article.
