# Supplementary material for: The Association Between Affect and Adiposity in Childhood and Adolescence: A Systematic Review
Source: Obes Rev. 2025 Dec 22;27(5):e70056. doi: 10.1111/obr.70056 (PMC13070898; doi:10.1111/obr.70056)
Supplement: Supplementary file 1 — Data S1: Supporting Information [file OBR-27-e70056-s001.pdf]

Supporting Information: *The association between affect and adiposity in childhood and adolescence: a systematic review*

Emma S. Young[1], Alice R. Kininmonth[2], Yue Wang[1], Jason CG Halford[1], Clare Llewellyn[3], Alison Fildes[1]

[1] School of Psychology, Faculty of Medicine and Health, University of Leeds, Leeds, UK

[2]School of Food Science and Nutrition, Faculty of Environment, University of Leeds, Leeds, UK

[3]Research Department of Behavioural Science, Institute of Epidemiology and Health Care, University College London, London, UK

Content included in this file:

Files S1, S1.1-S1.6: Search strategies for databases……………………………….**pp 2-8**

File S2: Summary of key search terms by PEO elements……………………………..**pp 9**

Files S3, S3.1- S3.6: Justification of inclusion for multiple reports……………………**pp 10-12**

File S4: Assessment of quality of included studies, based on an adapted Newcastle-Ottawa Scale (Modesti et al., 2016)…………………………………………**pp 13-18**

Table S1: Table of included longitudinal studies (n = 23)……………………………..**pp 19-20**

Table S2: Table of included non-longitudinal studies (n = 105)……………………….**pp 21-28**

File S5: Summary of studies examining associations using simple correlations……**pp 29-30**

File S1- Search strategies for databases

File S1.1 *OVID Medline search strategy*

1. exp Child/ or exp Adolescent/
2. (baby or babies or infan* or toddler* or child*).tw.
3. (paediatr* or pediatr*).tw.
4. (girl* or boy* or youth*).tw.
5. (schoolchild* or school child* or school-child*).tw.
6. (preschool* or pre-school*).tw.
7. young people.tw.
8. (preadolescen* or pre-adolescen*).tw.
9. (17 year old* or age 17).tw.
10. exp Affect/
11. exp Emotions/
12. exp Temperament/
13. (hedonic ajd2 (happiness or wellbeing)).tw.
14. (happy or happiness).tw.
15. (negative affect* or positive affect*).tw.
16. flourishing.tw.
17. (wellbeing adj2 (psychological or subjective or emotion*)).tw.
18. (emotion adj2 (problem* or difficult* or regulat* or dysregulat*)).tw.
19. (affect* adj2 (problem* or difficult* or regulat* or dysregulat*)).tw.
20. exp Obesity/ or exp Pediatric Obesity/ or exp Body weight/ or exp body mass index/ or exp overweight/
21. (BMI z-scores or BMI-for-age or weight-for-length or weight-for-height or waist circumference or body fat percentages or weight-for-age).tw.
22. (weight status or adipos*).tw.
23. (anthropometr* or growth charts).tw.
24. (underweight or under weight).tw.
25. (weight or BMI).tw.
26. (weight) adj2 (gain or loss or change)).tw.
27. 1 or 2 or 3 or 4 or 5 or 6 or 7 or 8 or 9
28. 10 or 11 or 12 or 13 or 14 or 15 or 16 or 17 or 18 or 19
29. 20 or 21 or 22 or 23 or 24 or 25 or 26
30. 27 and 28 and 29
31. Limit 30 to (English language and humans and journal article)

Results returned- 5761

File S1.2 *OVID APA PSYCHINFO search strategy*

1. (baby or babies or infan* or toddler* or child* or adolescen*).tw.
2. (paediatr* or pediatr*).tw.
3. (girl* or boy* or youth*).tw.
4. (schoolchild* or school child* or school-child*).tw.
5. (preschool* or pre-school*).tw.
6. young people.tw.
7. (17 year old* or age 17).tw.
8. exp Preadolescence/
9. exp Emotions/ or exp Affect Regulation/ or exp Emotional Regulation/ or exp Infant Temperament/
10. hedonic happiness.tw.
11. hedonic wellbeing.tw.
12. (happy or happiness).tw.
13. (negative affect* or positive affect*).tw.
14. flourishing.tw.
15. (wellbeing adj2 (psychological or subjective wellbeing or emotion*)).tw.
16. (emotion* adj2 (problem* or difficult*)).tw.
17. or (affect adj2 (problem* or difficult*)).tw.
18. exp Obesity/ or exp Body Mass Index/ or exp Body Weight/ or exp Overweight/ or exp Underweight/
19. (BMI z-scores or BMI-for-age or weight-for-length or weight-for-height or waist circumference or body fat percentages or weight-for-age).tw.
20. (weight status or adipos*).tw.
21. (anthropometr* or growth charts).tw.
22. (weight or BMI).tw.
23. (weight adj2 (gain or loss or change)).tw.
24. 1 or 2 or 3 or 4 or 5 or 6 or 7 or 8
25. 9 or 10 or 11 or 12 or 13 or 14 or 15 or 16 or 17
26. 18 or 19 or 20 or 21 or 22 or 23
27. 24 and 25 and 26
28. Limit 27 to (human and English language and “0110 peer-reviewed journal”)

Results returned- 1969

File S1.3 *OVID Embase search strategy*

1. exp Child/ or exp Adolescent/
2. (baby or babies or infan* or toddler* or child*).tw.
3. (paediatr* or pediatr*).tw.
4. (girl* or boy* or youth*).tw.
5. (schoolchild* or school child* or school-child*).tw.
6. (preschool* or pre-school*).tw.
7. young people.tw.
8. (preadolescen* or pre-adolescen*).tw.
9. (17 year old* or age 17).tw.
10. exp Emotions/ or exp emotional regulation/ or exp affect/
11. exp temperament/
12. hedonic happiness.tw.
13. hedonic wellbeing.tw.
14. (happy or happiness).tw.
15. (negative affect* or positive affect*).tw.
16. flourishing.tw.
17. (wellbeing adj2 (psychological or subjective or emotion*)).tw.
18. (emotion adj2 (problem* or difficult*)).tw.
19. (affect* adj2 (problem* or difficult* or regulat* or dysregulat*)).tw.
20. exp child obesity/ or exp adolescent obesity/ or exp obesity
21. exp underweight/ep, et, pc, rh, th [Epidemiology, Etiology, Prevention, Rehabilitation, Therapy]
22. (BMI z-scores or BMI-for-age or weight-for-length or weight-for-height or waist circumference or body fat percentages or weight-for-age).tw.
23. (body mass index or weight status or adipos*).tw.
24. (anthropometr* or growth charts).tw.
25. (weight or BMI).tw.
26. (weight) adj2 (gain or loss or change)).tw.
27. 1 or 2 or 3 or 4 or 5 or 6 or 7 or 8 or 9
28. 10 or 11 or 12 or 13 or 14 or 15 or 16 or 17 or 18 or 19
29. 20 or 21 or 22 or 23 or 24 or 25 or 26
30. 27 and 28 and 28
31. Limit 30 to (human and English language and article and journal)

Results returned- 6410

File S1.4 *Pubmed search strategy*

1. "Child"[mesh] OR "Adolescent"[mesh]
2. ("baby"[tiab] OR "babies"[tiab] OR infan*[tiab] OR toddler*[tiab] OR child*[tiab])
3. (paediatr*[tiab] OR pediatr*[tiab])
4. ("girl"[tiab] OR "girls"[tiab] OR "boy"[tiab] OR "boys"[tiab] OR youth*[tiab])
5. (schoolchild*[tiab] OR school child*[tiab] OR school-child*[tiab])
6. (preschool*[tiab] OR pre-school*[tiab])
7. "young people"[tiab]
8. (preadolescen*[tiab] OR pre-adolescen*[tiab])
9. (17 year old*[tiab] OR "age 17"[tiab])
10. "Affect"[mesh]
11. "Emotions"[mesh]
12. "Temperament"[mesh]
13. ("hedonic" AND ("happiness"[tiab] OR "wellbeing"[tiab]))
14. ("happy"[tiab] OR "happiness"[tiab])
15. (negative affect*[tiab] OR positive affect*[tiab])
16. "flourishing"[tiab]
17. ("wellbeing"[tiab] AND ("psychological"[tiab] OR "subjective"[tiab] OR emotion*[tiab]))
18. ("emotion"[tiab] AND (problem*[tiab] OR difficult*[tiab] OR regulat*[tiab] OR dysregulat*[tiab]))
19. (affect*[tiab] AND (problem*[tiab] OR difficult*[tiab] OR regulat*[tiab] OR dysregulat*[tiab]))
20. "Obesity"[mesh] OR "Pediatric Obesity"[mesh] OR "Body weight"[mesh] OR "body mass index"[mesh] OR "overweight"[mesh]
21. ("BMI z-scores"[tiab] OR "BMI-for-age"[tiab] OR "weight-for-length"[tiab] OR "weight-for-height"[tiab] OR "waist circumference"[tiab] OR "body fat percentages"[tiab] OR "weight-for-age"[tiab])
22. ("weight status"[tiab] OR adipos*[tiab])
23. (anthropometr*[tiab] OR "growth charts"[tiab])
24. ("underweight"[tiab] OR "under weight"[tiab])
25. ("weight"[tiab] OR "BMI"[tiab])
26. ("weight"[tiab]) AND ("gain"[tiab] OR "loss"[tiab] OR "change"[tiab]))
27. #1 OR #2 OR #3 OR #4 OR #5 OR #6 OR #7 OR #8 OR #9
28. #10 OR #11 OR #12 OR #13 OR #14 OR #15 OR #16 OR #17 OR #18 OR #19
29. #20 OR #21 OR #22 OR #23 OR #24 OR #25 OR #26
30. #27 AND #28 AND #29

Additional limitations: English language, Humans

Results returned- 7936

File S1.5 *Web of Science search strategy*

1. TS=Child
2. TS=Adolescen*
3. TS=(baby or babies or infan* or toddler* or child*)
4. TS=(paediatr* or pediatr*)
5. TS=(girl* or boy* or youth*)
6. TS=(schoolchild* or school child* or school-child*)
7. TS=(preschool* or pre-school*)
8. TS=(young people)
9. TS=(preadolescen* or pre-adolescen*)
10. TS=(17 year old* or age 17)
11. TS=(Emotions)
12. TS=(Temperament)
13. TS=(hedonic NEAR/2 (happiness or wellbeing))
14. TS=(happy or happiness)
15. TS=(negative affect* or positive affect*)
16. TS=(flourishing)
17. TS=(wellbeing NEAR/2 (psychological or subjective or emotion*))
18. TS=(emotion* NEAR/2 (problem* or difficult* or regulat* or dysregulat*))
19. TS=(affect* NEAR/2 (problem* or difficult* or regulat* or dysregulat*))
20. TS=(Obesity)
21. TS=(Pediatric Obesity)
22. TS=(Body weight)
23. TS=(Body mass index)
24. TS=(overweight)
25. TS=(BMI z-scores or BMI-for-age or weight-for-length or weight-for-height or waist circumference or body fat percentages or weight-for-age)
26. TS=(weight status or adipos*)
27. TS=(anthropometr* or growth charts)
28. TS=(underweight or under weight)
29. TS=(weight or BMI)
30. TS=(weight NEAR/2 (gain or loss or change))
31. #1 or #2 or #3 or #4 or #5 or #6 or #7 or #8 or #9 or #10
32. #11 or #12 or #13 or #14 or #15 or #16 or #17 or #18 or #19
33. #20 or #21 or #22 or #23 or #24 or #25 or #26 or #27 or #28 or #29 #or #30
34. #31 AND #32 AND #33 and Article (Document Types) and English (Languages)

Results returned- 8746

File S1.6 *EBSCO CINHAL search strategy*

1. (MH “Child+”)
2. (MH “Adolescence+”)
3. AB (baby or babies or infan* or toddler* or child*)
4. TI (baby or babies or infan* or toddler* or child*)
5. AB (paediatr* or pediatr*)
6. TI (paediatr* or pediatr*)
7. AB (girl* or boy* or youth*)
8. TI (girl* or boy* or youth*)
9. AB (schoolchild* or school child* or school-child*)
10. TI (schoolchild* or school child* or school-child*)
11. AB (preschool* or pre-school*)
12. TI (preschool* or pre-school*)
13. AB (young people)
14. TI (young people)
15. AB (preadolescen* or pre-adolescen*)
16. TI (preadolescen* or pre-adolescen*)
17. AB (17 year old* or age 17)
18. TI (17 year old* or age 17)
19. (MH “Emotions+”)
20. (MH “Temperament+”)
21. AB (hedonic N2 (happiness or wellbeing))
22. TI (hedonic N2 (happiness or wellbeing))
23. AB (happy or happiness)
24. TI (happy or happiness)
25. AB (negative affect* or positive affect*)
26. TI (negative affect* or positive affect*)
27. AB (flourishing)
28. TI (flourishing)
29. AB (wellbeing N2 (psychological or subjective or emotion*))
30. TI (wellbeing N2 (psychological or subjective or emotion*))
31. AB (emotion N2 (problem* or difficult* or regulat* or dysregulat*))
32. TI (emotion N2 (problem* or difficult* or regulat* or dysregulat*))
33. AB (affect N2 (problem* or difficult* or regulat* or dysregulat*))
34. TI (affect N2 (problem* or difficult* or regulat* or dysregulat*))
35. (MH “Obesity+”)
36. (MH “Pediatric obesity+”)
37. (MH “Body weight+”)
38. (MH “Body mass index+”)
39. AB (BMI z-scores or BMI-for-age or weight-for-length or weight-for-height or waist circumference or body fat percentages or weight-for-age))
40. TI (BMI z-scores or BMI-for-age or weight-for-length or weight-for-height or waist circumference or body fat percentages or weight-for-age))
41. AB (weight status or adipos*)
42. TI (weight status or adipos*)
43. AB (anthropometr* or growth charts
44. TI (anthropometr* or growth charts
45. AB (underweight or under weight)
46. TI (underweight or under weight)

47. AB (weight or BMI)
48. TI (weight or BMI)
49. AB (weight N2 (gain or loss or change))
50. TI (weight N2 (gain or loss or change))
51. (S1 OR S2 OR S3 OR S4 OR S5 OR S6 OR S7 OR S8 OR S9 OR S10 OR S11 OR S12 OR S13 OR S14 OR S15 OR S16 OR S17 OR S18)) AND (S1 OR S2 OR S3 OR S4 OR S5 OR S6 OR S7 OR S8 OR S9 OR S10 OR S11 OR S12 OR S13 OR S14 OR S15 OR S16 OR S17 OR S18)
52. (S19 OR S20 OR S21 OR S22 OR S23 OR S24 OR S25 OR S26 OR S27 OR S28 OR S29 OR S30 OR S31 OR S32 OR S33 OR S34)
53. (S35 OR S36 OR S37 OR S38 OR S39 OR S40 OR S41 OR S42 OR S43 OR S44 OR S45 OR S46 OR S47 OR S48 OR S49 OR S50)
54. (S51 AND S52 AND S53)

Additional Limits- English language, journal article, human subjects

Results returned- 2745

File S2: Summary of key search terms by PEO elements

**Population**		**Affect**		**Adiposity**	
Child/<br>Adolescent/<br>Baby<br>Babies<br>Infan*<br>Toddler*<br>Child*<br>Paediatr*<br>Pediatr*<br>Girl*<br>Boy*	Youth*<br>Schoolchild*<br>School child*<br>School-child*<br>Preschool*<br>Pre-school*<br>Young people<br>Preadolescen*<br>Pre-<br>adolescen*<br>17 year old*<br>Age 17	Affect/<br>Emotions/<br>Temperament/<br>Hedonic<br>Happiness<br>Hedonic<br>Wellbeing<br>Happy<br>Happiness<br>Negative<br>affect*<br>Positive affect*<br>Flourishing	Psychological<br>wellbeing<br>Subjective<br>wellbeing<br>Emotion*<br>wellbeing<br>Emotion*<br>problem*<br>Emotion*<br>difficult*<br>Emotion*<br>regulat*<br>Emotion*<br>dysregulat*<br>Affect* problem*<br>Affect* difficult*<br>Affect regulat*<br>Affect<br>dysregulat*	Obesity/<br>Pediatric<br>Obesity/<br>Body weight/<br>Body mass<br>index/<br>Overweight/<br>Underweight<br>Under weight<br>Weight<br>BMI<br>BMI z-scores<br>BMI-for-age<br>Weight-for-<br>length<br>Weight-for-<br>height	Weight-for-age<br>Waist circumference<br>Body fat percentages<br>Weight status<br>Adipos*<br>Anthropometr*<br>Growth charts<br>Weight gain<br>Weight loss<br>Weight change

File S3- Justification of inclusion for multiple reports

Where multiple studies reported associations within the same cohort, a systematic approach to selecting the study for inclusion was taken. In the first instance, longitudinal studies where directionality was tested were retained as a priority, favouring rigorous longitudinal methods. In the second instance, studies with the broadest range of time points (**TPs**) were retained, followed by highest sample size. If two studies had crossover, but the less methodologically 'rigorous' study provided a unique finding this was also retained, but only the findings from the unique analysis were reported. Tables S3.1 - S3.6 show the studies' characteristics, detail the decision to retain/exclude and provide a justification for the decision.

S3.1: *Millenium Cohort Study (MCS)- general negative affect*

Authors	Affect measure	Sample size	TPs	Statistical method and rigor	Decision	Justification
Patalay and Hardman, 2019	SDQ	17215	**5:** 3y, 5y, 7y, 11y, 14y	- Longitudinal<br>- CLPM<br>- Directionality tested	Retain	Most comprehensive span of analysis (11 years). Significantly higher sample size.
Kelly at al., 2016	SDQ, unhappiness (age 11)	16936	**4:** 3y, 5y, 7y, 11y	-Non longitudinal<br>-Logistic regression<br>- Directionality not tested	Retain, only report unhappiness	SDQ duplication of Patalay & Hardman and Creese et al., however unhappiness is a distinct and unique measurement- retained.
Griffiths et al., 2011[1]	SDQ	11202	**2:** 3y, 5y	-Linear and multinomial regression	Exclude	Shorter, less comprehensive analytic span. No unique measure to justify retention.
Creese et al., 2023	SDQ,	12450	**3:** 11y, 14y, 17y	-CPLM<br>-Directionality tested<br>-Mediation analysis	Retain	Extends beyond Patalay and Hardman into 17 years. Provides data on mediators, and sufficient distinction to justify retention.
Noonan & Fairclough, 2019	SDQ	6011	**1:** 7y	-Linear regression<br>-Moderators explored (gender, activity level)	Retain	Analysis presented with information of moderators not detailed elsewhere.

S3.2: *Millenium Cohort Study (MCS)- emotional regulation*

Authors	Affect measure	Sample size	TPs	Statistical method and rigor	Decision	Justification
Dos Santos et al., 2020	CSBQ	17165	**5:** 3y, 5y, 7y, 11y, 14y	Growth mixture modelling -Logistic Regression	Retain	More comprehensive time points, larger sample size.
Anderson et al., 2017[2]	CSBQ	10995	**4:** 3y, 5y, 7y, 11y	-Logistic Regression	Exclude	Fewer time points and smaller sample size.

S3.3: *Longitudinal Study of Australian children (LSAC)- general negative affect*

Authors	Affect measure	Sampl e size	TPs	Statistical method and rigor	Decision	Justification
Black and Kassen-boehmer, 2017	SDQ	4983	**5:** 4-5yr, 6-7y,8-9y,10-11y,12-13	-Longitudinal -Group differences -No directionality tested	Exclude	Studies testing directionality prioritised in first instance
Jansen et al.,	SDQ	1066*	**4:** 4-5y, 6-7y, 8-9y, 9-10y	-Longitudinal -Logistic regression -Directionality tested	Retain	Most comprehensive methodology and design
Sawyer et al., 2011[3]	SDQ	3363	**2:** 4-5yr, 8-9yr	-Longitudinal -Directionality tested	Exclude	Fewer time points, despite larger sample size

*Sensitivity analysis to control/adjust for baseline

S3.4: *Korean at Risk Youth Behaviour/KAHBOS survey- happiness*

Authors	Affect measure	Sample size	TPs	Statistical method and rigor	Decision	Justification
Moon and Kwon, 2020[4]	Single item happiness	62,276	**1:** (12-18y)	-T-tests -Weight dichotomised to Uw/Nw and Ow/Ob	Exclude	Smaller sample, less sensitive to weight spectrum
Min et al., 2017	Single item happiness	370,568	**1:** (12-18y)	-Logistic Regression -Wider selection of weight categories	Retain	Rigorous statistical approach, better sensitivity to weight spectrum

S3.5: *Children's body composition and stress study (ChIBS)- negative affect and happiness*

Authors	Affect measure	Sample size	TPs	Statistical method and rigor	Decision	Justification
Michels et al., 2015	SDQ, negative emotions	354	**3:** 5-10y, 10-117, 11-12y	-Longitudinal<br>-CLPM<br>-Directionality tested	Retain	Rigorous statistical approach
Vanalest et al., 2014	SDQ, negative emotions, happiness	355	**1:** 5-10y	-Cross-sectional<br>-Linear Regression	Retain	Study offers unique cross-sectional findings

S3.6: *[No cohort name]. Study of preschoolers in USA*

Authors	Affect measure	Sample size	TPs	Statistical method and rigor	Decision	Justification
Power et al., 2016	DoG task	187	**1:** (M= 57.4 mo)	-Cross-sectional<br>-DoG tasks explored by type<br>-Directionality not tested	Retain	Study offers marginally more detailed analysis
Hughes et al., 2015[5]	DoG task	187	**1:** (M= 57.4 mo)	-Cross-sectional<br>-DoG by wait-time<br>-Directionality not tested	Exclude	Study offers less detailed analysis

References (File S3)

Note: Only excluded studies (and not otherwise cited in-text of the main manuscript) are cited below.

1. L. J. Griffiths, C. Dezateux, and A. Hill, "Is Obesity Associated with Emotional and Behavioural Problems in Children? Findings from the Millennium Cohort Study," *International Journal of Pediatric Obesity* 6, no. 2 (2011): e423-432, https://doi.org/10.3109/17477166.2010.526221.
2. S. E. Anderson, A. Sacker, R. C. Whitaker, and Y. Kelly, "Self-Regulation and Household Routines at Age Three and Obesity at Age Eleven: Longitudinal Analysis of the UK Millennium Cohort Study", *International Journal of Obesity (London)* 41, no. 10 (2017): 1459-1466, https://doi.org/10.1038/ijo.2017.94.
3. M. G. Sawyer, T. Harchak, M. Wake, and J. Lynch, "Four-Year Prospective Study of BMI and Mental Health problems in Young Children," *Pediatrics* 128, no. 4 (2011): 677-684, https://doi.org/10.1542/peds.2010-3132.
4. W. H. Moon, and M. Kwon, "Major Factors Having Influence on the Subjective Happiness of Korean Youth; From the 13th KAHBOS Data," *Medico-Legal Update* 20 (2020): 1564-1570.
5. S. O. Hughes, T. G. Power, T. M. O'Connor and J. O. Fisher, "Executive Functioning, Emotion Regulation, Eating Self-Regulation, and Weight Status in Low-Income Preschool Children: How Do They Relate?," *Appetite* 89 (2015): 1-9, https://doi.org/10.1016/j.appet.2015.01.009.

File S4: Assessment of quality of included studies in the Affect x Adiposity systematic review, based on the adapted Newcastle-Ottawa Scale for cross-sectional designs (Modesti et al., 2016)

	Selection				Comparability		Outcome		
**Authors, year**	**Representativeness of sample**	**Sample Size**	**Non-respondents**	**Ascertainment of affect**	**Most important confounder**	**Additional confounders**	**Ascertainment of adiposity**	**Statistical quality**	**Score**
Abdel-Aziz et al., 2014	C	B*	C	B*	A*	A*	A**	A*	7
Aditya & Sekartini, 2017	B*	C	B	B*	B	B	A**	B	4
Alexius et al., 2012	B*	A*	C	A**/B*[1]	A*	A*	A**	A*	9/8
Alves et al.,2021	C	C	B	B*	A*	A*	A**	A*	6
Biggs et al., 2017	C	C	C	B*	B	B	E	A*	2
Bjertnaes et al., 2020	B*	B*	B	A**	A*	A*	C	A*	7
Blanco et al., 2020	C	B*	B	A**	A*	A*	A**	A*	7
Brinksma et al., 2015	C	B*	A*	B*	A*	A*	A**	A*	8
Button et al., 2021	D	C	C	A**	A*	A*	A**	A*	7
Byrne et al., 2023	B*	B*	C	A**	A*	A*	A**	B	8
Çalişkan & Özyurt, 2020	B*	C	C	B*	B	B	A**	A*	5
Carey, 1985	B*	C	C	B*	B	B	A**	A*	5
Chan & Wang, 2013	B*	C	C	A**	A*	A*	D[2]	A*	6
Çolpan et al., 2018	C	B*	C	A**	A*	A*	A**	A*	8
Creese et al., 2023	A*	B*	B	A**	A*	A*	A**	A*	9
Crewther et al., 2024	C	B*	C	B*	A*	A*	A**	A*	7
Czepczor-Bernat et al., 2020	B*	C	C	A**	B	B	C/D[3]	B	3
Darlington & Wright, 2006	B*	C	C	A**	A*	A*	A**	A*	8
Donkor et al., 2021	B*	B*	A*	A**	A*	A*	A**	A*	10
Donnchadha et al., 2023	A*	B*	B	A**	A*	A*	A**	A*	9
Doom et al., 2023	B*	B*	A*	B*	A*	A*	A**/C[4]	A*	9/7
Dos Santos et al., 2020	A*	B*	A*	A**	B	A*	A**	A*	10
Drukker et al., 2009	B*	B*	C	B*	A*	A*	B**	A*	8
Eiffener et al., 2019	C	C	C	B*	B	A*	A**	A*	6

Faith & Hittner, 2010	B*	B*	C	B*	A*	A*	A**/B**[5]	A*	7
Farajpour et al., 2018	B*	B*	C	B*	B	B	A**	A*	6
Farrow et al., 2018	B*	C	A*	A**	A*	A*	A**	A*	9
Fonseca et al., 2009	A*	B*	C	B*	B	B	C	A*	4
Förster et al., 2023	B*	B*	C	B*	A*	A*	A**	A*	7
Frerichs et al., 2020	C	C	A*	A**	A*	A*	A**/B**[5]	A*	8
Gandhi et al., 2015	A*	B*	B	A**	B	B	D	A*	5
Giacomo et al., 2019	B*	B*	C	B*	B	B	E	B	3
Gil-Madrona et al., 2019	B*	B*	C	A**	B	B	A**	A*	7
Gopinath et al., 2013	A*	B*	B	B*	A*	A*	A**	A*	8
Gowey et al., 2014	C	C	C	A**	B	A*	A**	A*	6
Grammer et al., 2018	B*	C	C	A**	A*	B	A**	B	6
Graziano et al., 2013	B*	B*	A*	A**	B	A*	A**	A*	9
Graziano et al., 2010	B*	B*	B	A**	B	A*	A**	A*	8
Hainsworth et al., 2009	C	B*	C	A**	B	B	A**	A*	6
Hallal et al., 2010	B*	B*	C	B*	A*	A*	E	A*	6
Hampel et al., 2021	C	B*	C	A**	A*	A*	A**	A*	8
Hanć et al., 2014	B*	A*	C	A**	B	A*	A**	A*	8
Hankey et al., 2017	B*	B*	A*	A**	A*	A*	A**	A*	10
Harcourt et al., 2019	C	B*	A*	B*	A*	A*	A**	A*	8
Haycraft et al., 2011	B*	C	C	A**	A*	B	C/D	A*	5
Hillman et al., 2010	C	C	C	A**	A*	A*	A**	A*	7
Hoare et al., 2019	B*	B*	B	A**	A*	A*	A**	A*	9
Hughes et al., 2008	B*	B*	B	A**	A*	A*	A**	A*	9
Hughes et al., 2007	C	A*	B	A**	A*	A*	A**	A*	8
Innella et al., 2019	B*	C	C	A**	A*	A*	A**	A*/B[6]	9/8
Ivarsson et al., 2006	B*	B*	C	B*	A*	A*	C	A*	6
Jansen et al., 2008	B*	B*	A*	A**	A*	A*	A**	A*	10
Jansen et al., 2013	A*	B*	A*	A**	A*	A*	A**	A*	10
Jeffery et al., 2014	B*	C	C	A**/B*[1]	A*	A*	A**	A*	8/7

Johnson & Greene, 1991	C	C	C	A**	B	B	A**	A*	5
Keating et al., 2011	B*	B*	C	A**	B	A*	A**	A*	8
Kelly et al., 2016[a]	A*	B*	B	A**	A*	A*	A**	A*	9
Kelly et al., 2016[b]	B*	C	C	A**	A*	A*	A**	A*	8
Kohlboeck et al., 2014	B*	B*	A*	B*	A*	A*	A**	B	8
Kong et al., 2019	C	B*	C	A**	A*	A*	A**	A*	8
Kong et al., 2022	B*	B*	C	A**	A*	A*	A**	A*	9
Kubzansky et al., 2012	B*	B*	A*	B*	A*	A*	A**	A*	9
Lee et al., 2020	A*	B*	C	A**	A*	A*	D	A*	7
Leung et al., 2016	C	C	C	A**	A*	B	A**	B	5
Li et al., 2007	B*	B*	C	A**	B	A*	A**	A*	7
Liew et al., 2020	B*	C	C	A**	A*	A*	A**	B	7
Liu et al., 2023	C	B*	C	A**	A*	A*	B**/D[7]	A*	8/6
Liu et al., 2016	B*	B*	B	B*	A*	A*	A**	A*	8
Loh et al., 2015	A*	A*	B	A**	A*	A*	A**	A*	9
Mallan et al., 2017	B*	C	A*	A**	A*	A*	A**	B	8
McCabe et al., 2005	B*	B*	A*	A**	A*	B	A**	A*	9
Melis Yavuz et al., 2018	C	B*	C	A**	B	A*	A**	A*	7
Michels et al., 2015	B*	C	A*	A**	A*	A*	A**	A*	9
Midei &Matthews, 2009	B*	C	A*	A**	A*	A*	A**	A*	9
Miller et al., 2016	C	B*	C	A**	A*	A*	A**	A*	8
Min et al., 2017	A*	B*	C	A**	A*	A*	D	A*	7
Miri et al., 2017	B*	C	C	A**	B	A*	A**	B	6
Morrison et al., 2015	C	B*	C	B*	A*	A*	A**	A*	7
Mota et al., 2018	C	A*	B	A**	B	B	A**	A*	6
Noonan & Fairclough, 2019	A*	B*	C	B*	A*	A*	A**	A*	8
Ohrt et al., 2023	B*	B*	C	A**	A*	A*	A**	A*	9
Orihuela et al., 2017	C	C	A*	A**	A*	A*	A**	A*	8
Öz & Kivrak, 2023	C	A*	C	A**	B	A*	A**	A*	7
Özyurt et al., 2022	C	A*	C	A**	A*	A*	A**	A*	8

Pace et al., 2019	C	B*	C	A**	A*	A*	B**/E[8]	A*	8/6
Pan et al., 2018	C	C	C	B*	B	B	E	A*	2
Patalay & Hardman, 2019	A*	B*	C	B*	A*	A*	A**	A*	8
Percinel et al., 2018	C	C	C	A**	B	A*	B**	A*	6
Pérez-Bonaventura et al., 2015	B*	B*	A*	A**	A*	B	A**	A*	9
Perez-Sousa et al., 2018	C	B*	C	B*	B	B	A**	A*	5
Petersen et al., 2014	B*	B*	B	A**	B	A*	A**	A*	8
Pitrou et al., 2010	A*	B*	B	B*	A*	A*	D	A*	6
Power et al., 2020	C	B*	C	A**	B	A*	A**	A*	7
Rajan et al., 2019	B*	C	C	A**	A*	A*	A**	A*	8
Reinelt et al., 2020	C	B*	C	A**	A*	A*	A**	A*	8
Ren et al., 2018	B*	B*	C	A**	A*	A*	A**	A*	9
Rhee et al., 2021	B*	C	C	A**	A*	A*	A**	A*	8
Riahi et al., 2019	A*	B*	B	A**	B	A*	A**	A*	8
Riazi et al., 2010	B*	B*	B	A**	A*	A*	A**	B	8
Rosenthal et al., 2015	B*	B*	C	A**	A*	A*	A**	B	8
Sahin & Kirli, 2021	C	B*	C	A**	B	A*	C	A*	5
Santos et al., 2023	B*	A*	C	B*	B	B	E	A*	4
Selewski et al., 2013	C	B*	C	A**	B	A*	A**	A*	7
Sepúlveda et al., 2020	C	B*	C	A**	A*	A*	A**	A*	8
Shinsugi et al., 2021	B*	B*	B	A**	A*	A*	A**	A*	9
Shriver et al., 2019	B*	C	C	A**	A*	A*	A**	A*	8
Sinclair et al., 2016	B*	A*	C	B*	A*	A*	A**	A*	8
Steen et al., 1996	B*	B*	C	B*	B	B	A**	B	5
Stifter and Moding, 2018	B*	C	A*	A**	A*	A*	A**	A*	9
Stifter et al., 2011	B*	C	B	A**	A*	A*	A**	A*	8
Stival et al., 2022	B*	A*	C	B*	B	A*	D	A*	5
Suchert et al., 2016	B*	B*	B	A**	A*	A*	A**	B	8
Sutin et al., 2021	D	B*	C	A**	A*	A*	A**	A*	8

Topçu et al., 2016	C	B*	C	A**	B	A*	A**	A*	7
van der Voorn et al., 2023	C	B*	C	A**	A*	A*	A**	A*	8
van Grieken et al., 2013	A*	B*	B	B*	B	A*	A**	A*	7
Vanaelst et al., 2014	B*	C	A*	B*	A*	A*	A**	A*	8
Vaquero-Soliś et al., 2021	B*	B*	C	A**	B	B	A**	B	6
Vila et al., 1995	C	C	C	B*	B	B	E	A*	2
Vollrath et al., 2018	A*	B*	B	B*	A*	A*	B**/D[9]	B	7/5
Ward-Begnoche et al., 2011	C	C	C	A**	B	B	A**	C	4
Williams et al., 2011	B*	B*	A*	B*	A*	A*	A**	A*	9
Williams et al., 2005	B*	A*	C	A**	B	A*	A**	A*	8
Wood et al., 2022	B*	B*	C	A**	A*	A*	A**	A*	9
Yackobovitch-Gavan et al., 2008	C	A*	A*	A**	B	B	A**	A*	7
Yilmaz Kafali et al., 2021	C	A*	C	A**	B	B	A**	B	5
Zhao et al., 2019	A*	B*	C	A**	A*	A*	D	A*	7
Zuba & Warschburger, 2017	B*	B*	C	A**	A*	A*	A**	A*	9

[a] Kelly et al. (2016)- Y. Kelly, P. Patalay, S. Montogomery, and A. Sacker
[b]Kelly et al. (2016)- N. R. Kelly, M. Tanofsky-Kraff, A. Vannucci, et al.
[1] Validation information derived from populations not included in the study
[2] Reported by teacher
[3]Self-reported but younger children were supported by parents to take measures
[4] Either self-reported or measured
[5]Either measured or obtained from records
[6]Cross-sectional data with partial correlations (appropriate) and mediational analysis (not appropriate)
[7]Parent reported and obtained from records
[8] Exposed cohort (OB) obtained from records, not specified for control group
[9] Extracted from record at birth, parent report of records thereafter

CRITERIA:
Selection (max 5 stars)
1) Representativeness of the exposed cohort
A*= Truly representative of the general population
B* = Somewhat representative of the general population
C = Selected group of users e.g. nurses, volunteers
D = No description of sampling strategy
2) Sample size§
A* = Justified and satisfactory
B* = Satisfactory, but not justified OR Not satisfactory, but justified
C = Not justified or satisfactory
3) Non-respondents: ¶
A* = Comparability between respondents and non-respondents characteristics is established, and the response rate is satisfactory
B = The response rate is unsatisfactory, or the comparability between respondents and non-respondents is unsatisfactory.

C = No description of the response rate or the characteristics of the responders and the non-responders
4) Ascertainment of exposure (affect)
A** = Self or parent-administered questionnaire (with extra validation/ reliability information reported for specific target sample)
B* = Parent or self-reported questionnaire
C = No description of the measurement tool

Comparability (max 2 stars)
1) The most important confounding factor is controlled (birth weight, puberty, age, sex, an indicator of socioeconomic status etc.) [1]
A* = Yes
B = No
2) The study control for any additional confounding factors.
A* = Yes
B = No

Outcome (max 3 stars)
1) Assessment of outcome (adiposity)[2]
A** = Clinical assessment
B** = Record linkage
C = Self-report
D = Parent-report
E= Not specified
2) Statistical test:
A* = The statistical test used to analyse the data is clearly described and appropriate, and the measurement of the association is presented, including confidence intervals and the probability level (p value)
B= The statistical test is not appropriate, not described or incomplete.

This tool is an adapted version of the Newcastle Ottawa Scale, to accommodate cross-sectional designs (Modesti et al., 2016). Amendments were made to this tool to reflect the context of the current review and included studies, as below:

§ The addition of 'Justified but not satisfactory' (B*) was to account for studies with very large sample sizes, where a justification for the sample size (i.e., a power calculation) was unnecessary.

B*= Studies that computed power calculation but did not meet thresholds for satisfactory sample size OR 'Satisfactory but not justified'- no power calculation but sufficiently large sample size, as below:

- Observational cohort, community studies: > 400 participants
- Clinical studies: >100 participants (or >50 in exposed cohort)
- Lab studies (infancy, affect observed directly): >100 participants

¶ Characteristics of non-respondents could constitute characteristics of loss-to follow up in prospective or longitudinal studies.

[1] Because of the variety of study and sample characteristics, no one confounding variable was considered the most important. Therefore, the most important confounding factor varied on a case-by-case basis, and could have included: Birthweight, pubertal status, age, sex, an indicator of socio-economic status, home chaos, treatment group.

[2] Added to account for studies where there was no clear description of how adiposity data was collected ('E').

Table S1: Table of included longitudinal studies (n = 23)

Authors	Year	N (%F), Age range /M(*SD*)[1]	FU (y)	Setting	Country	Affect measure	Adiposity measure	Association between affect and adiposity
Temperament Studies (n = 9)								
Carey	1985	200 (46%), 6mo	0.5	Community	USA	ITQ	WfL	Negative mood (i.e., more frequent crying and fussing) at ~6 months of age associated with greater WfL gain between 6-12months (t= 2.85, p =.006). No association between mood and WfL loss.
Faith & Hittner	2010	487 (46%), 1y	5	Community	USA	CCTI	WfL, BMI z-score[a]	No association between emotionality in year 1 with weight change from 1-6years, or weight status at age 6.
Farrow et al.	2018	62 (47%), 3-5y	2	Community	UK	EAS	BMI z-score[b]	Negative emotionality at 3-5 years associated with a lower BMI z-score at 5-7 years (partial r= -.53, p<.01).
Frerichs et al.	2018	68 (53%), 5-11y	0.5	Clinical	USA	CCTI	>95th centile[a]	No association between baseline emotionality and decrease in waist circumference, BF% or proportion of children > %95th BMI over treatment course.
Kong et al.	2019	216 (51%), 1mo	6	Community	USA	PCERA	BMI trajectory[c]	Positive affect during play associated with normal growth trajectory between infancy and mid childhood (β =.463, p=.006)
Liu et al.	2023	384 (NS) **, 4.5y	4.5	Community	USA	CBQ	BMI z-score[d]	Higher negative affectivity associated with less stability in BMI trajectory (β=.13, p<.017)
Stifter & Moding	2018	160 (47%), 6mo	0.5	Community	USA	IBQ	WfL z-score[d]	No association between negative affect or surgency at 6 months and WfL z-score gain between 6-12 months.
Rajan et al.	2019	204 (42%), 4y	1	Community	Mexico	CBQ-VSF	BMI[e]	No association between negative temperament at age 4 and BMI age 5.
Vollrath et al.	2018	25889 (49%), 1.5y	9	Community	Norway	EAS	BMI[c], adiposity rebound	No association between negative emotionality at age 1.5, 3 or 5 years and adiposity rebound.
Emotional Functioning Studies (n = 2)								
Harcourt et al.	2019	250 (52%), 2-18y	1	Clinical	Australia	PedsQL, SDQ	BMI z-score[a]	Greater baseline emotional functioning associated with higher likelihood of BMI z-score reduction 12 months later (OR= 1.32, p<.01). No association between emotional problems and weight change.
Yackobovitch-Gavan et al.	2008	71 (58%), 12-18y	1	Clinical	Israel	PedsQL	BMI SDS[a]	Greater baseline emotional functioning associated with a higher reduction in BMI-SDS between pre and post intervention (b= .016, p= .005).
Emotional Regulation studies (n = 4)								
Dos Santos et al.	2020	17,165 (49%), 3y	11	Community	UK	CSBQ, ITQ	BMI z-score[d]	No association between BMI z-score and emotional dysregulation. 'Early weight gain' trajectory membership associated with greater negative mood compared to 'Weight loss' trajectory (OR = 1.047, CI = 1.006-1.089). No other associations between mood and growth trajectory class membership.
Eiffener et al.	2019	77 (53%), 4-6y	1	Clinical	Sweden	CBCL	BMI z-score[f]	No association between baseline emotional reactivity and BMI z-score change pre-post intervention.
Graziano et al.	2010	57 (48%), 2y	3.5	Community	USA	LAB-TAB	Weight status[d]	Better emotional regulation at age 2 associated with smaller likelihood of BMI increase between ages 2 and 5.5 years (β = -.41, p< .01). Children classified as being or at risk of OW at age 5.5 had poorer emotional regulation skills at age 2, comparative to children classified as 'normal weight' (F(1,53) = 6.74, p<.05).

Authors	Year	N (%F), Age range /M(*SD*)[1]	FU (y)	Setting	Country	Affect measure	Adiposity measure	Association between affect and adiposity
Orihuela et al.	2017	79 (53%), 15y	1	Community	USA	ERICA	BMI centile[d]	Higher BMI centile at age 15 associated with better ER at age 16 (β = .26, p<.01).
Negative affect studies (n = 8)								
Jansen et al.	2008	787 (48%), 9-10y	3	Community	Netherlands	SDQ	Weight status[f]	No association between weight status at age 9-10 and emotional problems at age 12-13 or vice versa.
Jansen et al.	2013	3197 (49%) 4-5y	6	Community	Australia	SDQ	Weight status[f]	1 or 2 episodes of OW between 4/5y and 10/11y associated with significantly higher odds of above-threshold emotional problems at 10-11y, than 0 episodes (1-episode OR=1.41, p<.05; 2-episode OR 1.1, p <.05). Three or 4 episodes of OW not associated with emotional problems. No association between emotional problems above 'normal' threshold at 4-5y and OW at 10-11y.
Michels et al.	2015	316 (50%), 5-10y	3	Community	Belgium	SDQ, single items 'anxiety', 'sadness' and 'anger'	BMI z-score[g], BF%	Higher BMI and BF% at age 6-11years (T2) associated with greater negative emotions (T3) 1 year later (BMI β =.197, p =.04, BF% β = .169, p=.007). No association between negative emotions at 6-11years and BMI or BF% 1 year later. No association between adiposity and negative emotions in either direction between 5-10years (T1) and 6-11years (T2).
Midei &Matthews	2009	213 (51%), 14y	3	Community	USA	STAI, STAXI	WC, WHR, BMI[c]	Trait anger at age 14 associated with greater WHR at age 17 (β =.117, p = .05). WHR and trait anxiety associated cross-sectionally at age 14 (β= .139, p<.02), but not longitudinally.
Creese et al.	2023	12450 (48%), 11y	6	Community	UK	SDQ	BMI z-score[d]	BMI z-score at age 11 associated with greater emotional problems at age 17 for boys (OR = 1.09, p <.05), but no direct association between emotional problems at age 11 and BMI z-score at age 17. No direct associations in either direction for girls.
Patalay & Hardman	2019	17215 (49%), 3y	11	Community	UK	SDQ	BMI centile[f]	BMI at age 7 associated with greater emotional problems at age 11 (β = .019, p<.05), but emotional problems at age 7 not associated with BMI at age 11. Emotional problems at age 11 associated with greater BMI in at age 14 (β = .030, p<.05), but BMI at age 11 not associated with emotional problems at age 14. No association between emotional problems and BMI in early childhood (3-7years).
Pérez-Bonaventura et al.	2015	611 (50%), 3y	2	Community	Spain	SDQ	BMI z-score[a]	No association between BMI z-score at age 3 and emotional problems at age 4 or age 5 years.
Zuba & Warschburger	2017	1047 (52%), 7-11y	2	Community	Germany	SDQ	BMI SDS[h]	No direct association between BMI SDS at age 7 with emotional problems at age 11.

[1] Age at baseline. ** Sensitivity analysis- no available data on gender. **Abbreviations**: BF%- Body fat percentage; BMI- Body mass index; CBQ- Child Behaviour Questionnaire[1]; CBCL- Child Behaviour Checklist[2]; CCTI- Colorado Child Temperament Inventory[3]; CSBQ- Child Social Behaviour Questionnaire[4]; EAS- Emotionality, Activity and Sociability Scale[5]; ERC- Emotional Regulation Checklist[6]; ERICA- Emotional Regulation Index for Children and Adolescents[7]; ERQ- Emotional Regulation Questionnaire[8]; FU- Follow up period; IBQ- Infant Behaviour Questionnaire[9]; ITQ- Infant Temperament Questionnaire[10] NS- not specified; LAB-TAB – Laboratory Temperament Assessment Battery[11]; OB- Obesity; OW- Overweight; PCERA- Parent-Child Early Relational Assessment[12]; PEDS-QL- Paediatric Quality of Life[13]; SDS- Standard deviation score; SDQ- Strengths and Difficulties questionnaire[14]; STAI- State/Trait Anxiety Inventory[15]; STAXI- State/Trait Anger Expression Inventory[16]; UW- Underweight; WC- Waist circumference; WHtR- Waist-to-height-ratio; WfL- Weight-for-length.

[a] Centre for Disease Control (CDC, 2000) Growth Reference Charts
[b] Child Growth Foundation 1996
[c] None/Not Specified
[d] World Health Organisation (WHO) 2007
[e] National Institutes of Health 1991
[f] International Obesity Task Force (IOTF)
[g] UK Growth Reference 1990
[h] German population growth reference 2002

Table S2: Table of included non-longitudinal studies (n = 105)

Authors	Year	N (%F), age range/M(*SD*)[1]	Setting, design	Country	Affect Measure	Adiposity Measure	Association between affect and adiposity
Temperament Studies (n = 14)							
Button et al.	2021	283 (% NS), birth	Community, Prosp *(9-18mo FU*)	USA	IBQ	WfL z-score, WfA z-score[i]	No association between rapid weight gain between birth and 9-18 months, and infant negativity at 9-18 months.
Darlington & Wright	2006	75 (43%), 8wk	Community, Prosp *(4wk FU)*	UK	IBQ	Weight gain (g)	Slow weight gain between birth and 8weeks associated with fear at 8-12 weeks (b= -289.96 p<.01). Faster weight gain associated with negative emotionality (b= 217.05, p<.05). No association for smiling and laughter.
Graziano et al.	2013	195 (58%), 2y (24-45mo)	Community, Prosp *(8y FU)*	USA	TBAQ	BMI z-score[a]	Higher scores of 'pleasure' temperament at age 2 (e.g. laughing and smiling) associated with larger BMI z-score change between ages 4 and 10 (β= .13, p<.05).
Leung et al.	2016	379 (50%), 4.2 (.53)y	Community, Prosp *(2y FU)*	USA	CBQ, ERC	BMI z-score[a]	No direct association between surgency and concurrent BMI z-score. No association with rate of change in BMI-z-score. Negative lability not associated with concurrent BMI z-score or rate of change.
Hankey et al.	2017	180 (51%), 5.25-5.29y	Community, Prosp *(7y FU)*	USA	CBQ	BMI z-score[a]	Surgency in early childhood (~5y) associated with higher BMI z-score in middle childhood (~7-8y) (β= .19, p= .015) and adolescence (~12y) (β= .17, p<.05).
Wood et al.	2022	126 (52%), 4mo	Community, Prosp *(8mo FU)*	USA	LAB-TAB	BF%; WfL z-score, BMI z-score[d]	*Non-social positive affect* at 4 months associated with greater WfL z-score at 12 months (β= 1.49, p= .03), but not cross-sectionally. No association between non-social positive affect and other adiposity indices. *Social positive affect* at 4 months inversely associated with BF% at 12 months (β= -11.41, p= .04). No other association with indices of adiposity cross-sectionally or at 12 months.
Haycraft et al.	2011	139 (45%), 3-8y	Community, Cross-sec	UK	EAS	BMI[b]	No association between negative emotionality and BMI.
Innella et al.	2019	100 (<50%), 2-6y	Community, Cross-sec	USA	CBQ-SF	BMI z-score[a]	Surgency negatively associated with BMI z-score (partial r= -.21, p<.05). No direct association for negative affectivity.
Kong et al.	2022	121 (52%), 9-15 mo	Community, Cross-sec	USA	PCERA	WfL z-score[d]	Negative affect during play positively associated with greater WfL z-score (b= .383, p<.05).
Hughes et al.	2008	718 (48%), M= 4.40 (.60)y	Community, Cross-sec	USA	CBQ	BMI z-score[a]	No association between negative affectivity or surgency with BMI z-score.
Liew et al.	2020	221 (49%), 4-6y	Community, Cross-sec	USA	CBQ-SF	BMI z-score[a]	No direct association between negative affectivity and BMI.
Melis Yavuz & Selcuk	2018	122 (48%), 3-6y	Community, Cross-sec	Türkiye	CBQ	Weight status[f]	No association between weight status and negative affect. No group differences in negative affect.
Ohrt et al.	2023	220 (50%), 4-6y	Community, Cross-sec	USA	CBQ NA	BMI and BF% z-scores, summed[a]	No association between negative affectivity and adiposity.
Stifter et al.	2011	100 (57%), 3-34mo	Community, Cross-sec	USA	IBQ	BMI-for-age z-score[d]	Negative temperament negatively associated with BMI (B = -2.69, SE 1.26, p= .0367).
Emotional Functioning Studies (n = 25)							
Abdel-Aziz et al.	2014	100 (39%), 7-17y	Clinical, Cross-sec	Egypt	PedsQL	Weight status[f]	Self-reported and parent-reported emotional functioning lower in OB children than controls (SR t= 6.2, p= .020; PR t= 3.7, p= .01).

Authors	Year	N (%F), age range/M(*SD*)[1]	Setting, design	Country	Affect Measure	Adiposity Measure	Association between affect and adiposity
Hainsworth et al.	2009	319 (71%), 8-18y	Clinical, Cross-sec	USA	PedsQL	Weight status[a]	Self-reported emotional functioning lower in OB than OW children and adolescents (F= 4.6, p= .01). No differences in Ob vs NW children. No differences between any group in parent-reported functioning.
Hughes et al.	2007	126 (56%), M= 8.61(1.9)y	Clinical, Cross-sec	Scotland, UK	PedsQL	BMI centile[g]	OB group had lower parent-proxy emotional functioning than control group (p<.001). No difference with self-reported functioning.
Mota et al.	2018	150 (55%), 11-15y	Clinical, Cross-sec	Brazil	PedsQL	BMI z-score[d]	No difference in emotional functioning scores between OW and NW group.
Perez-Sousa et al.	2018	338 (49%), 6-14y	Clinical, Cross-sec	Spain	PedsQL	Weight status[f]	Emotional functioning scores lower in Ob/Ow children than NW (p <.05).
Riazi et al.	2010	540 (56%), 5-16y	Clinical, Cross-sec	UK	PedsQL	BMI z-score[b]	Emotional functioning lower in OB group than control group. BMI z-score associated with emotional functioning (p <.05).
van der Voorn et al.	2023	561 (61%), 5-19y	Clinical, Cross-sec	Netherlands	PedsQL	BMI SDS[f,j]	Emotional functioning lower in children with SOB compared to OW (p <.05). No differences between OW and OB, and OB and SOB groups.
Gopinath et al.	2013	2353 (61%), Med = 12.7y	Community, Prosp *(5yr FU)*	Australia	PedsQL	Weight status[f]	No difference between emotional functioning score at age 17 by weight status at age 12.
Williams et al.	2011	851 (50%), 8-13y	Community, Prosp (*5y FU)*	Australia	PedsQL	Weight status[a]	No association between weight status at age 8-13 and emotional functioning 5 years later.
Morrison et al.	2015	244 (51%), 8-17y	Community, Cross-sec	Canada	PedsQL	BF%, BMI z-score[a]	No association between emotional functioning and BMI z-score or BF%.
Hoare et al.	2019	809 (53%), 9-12y	Community, Cross-sec	Australia	PedsQL	Weight status[f]	No association between emotional functioning and OW/OB.
Loh et al.	2015	432(73%), 13y	Community, Cross-sec	Malaysia	PedsQL	Weight status[f]	No difference in emotional functioning scores by weight status.
Santos et al.	2023	181 (56%), 5-13y	Community, Cross-sec	Brazil	PedsQL	Weight status[d]	No difference in emotional functioning scores and weight status.
Brinksma et al.	2015	104 (54%), 2-18y	Clinical, Cross-sec	Netherlands	PedsQL	BMI SDS[j]	Overnourished (>2 SDS) children had worse emotional functioning scores than 'normal' weight children (p= .029).
Chan & Wang	2013	336 (47%), 2-7y	Community, Cross-sec	China	PedsQL	Weight status[f]	NW children more likely to score in lowest quartile for emotional functioning, relative to OB children (OR= .235, CI = .074-.743). No other differences in emotional functioning scores by weight category.
Farajpour et al.	2018	829 (56%), 8-12y	Community, Cross-sec	Iran	PedsQL	Weight status[a]	*Parent-reported: E*motional functioning scores worse in OB group than OW or NW (p= .045). *Self-reported*: No differences in emotional functioning by weight status.
Gandhi et al.	2015	323 (%), 15-18y	Community, Cross-sec	USA	PedsQL	Weight status[a]	No differences in emotional functioning by weight status.
Gowey et al.	2014	272 (%), 8-12y	Community, Cross-sec	USA	PedsQL	BMI z-score[a]	No association between BMI z-score and emotional functioning scores.
Keating et al.	2011	2890 (44%), 11-18y	Community, Cross-sec	Australia	PedsQL	Weight status[f]	OB group score worse on emotional functioning than NW counterparts (p= .004). No difference between OW and NW groups.
Lee et al.	2020	21,359 (51%), 12-13y	Community, Cross-sec	Across SE Asia[2]	PedsQL	Weight status[k]	No association between emotional functioning and weight status.
Liu et al.	2016	5781 (45%), 8-12y	Community, Cross-sec	China	PedsQL	Weight status, BMI z-score[d]	No association between emotional functioning and BMI z-score or weight status.
Miri et al.	2017	236 (48%), 12-18y	Community, Cross-sec	Iran	PedsQL	Weight status[d]	Worse emotional functioning associated with OW or OB (OR= .21, CI= .11-.32, p= .001).

Authors	Year	N (%F), age range/M(*SD*)[1]	Setting, design	Country	Affect Measure	Adiposity Measure	Association between affect and adiposity
Petersen et al.	2014	8947 (53%), 12-18y	Community, Cross-sec	Fiji	PedsQL	Weight status[f]	Emotional functioning higher in OB group compared to NW in adolescents aged 12-14 (p<.01), and lower than the NW group in adolescents aged 15-18 (p<.01).
Sinclair et al.	2016	7237 (53%), 15-17y	Community, Cross-sec	Fiji	PedsQL	BMI z-score[c]	*Age 15 (W1):* Emotional functioning associated with BMI z-score in boys (B= 0.7, p <.00). No association in girls. *Age 17 (W2):* Emotional functioning associated with BMI z-score in girls (B= 1.0, p= .03). No association in boys.
Williams et al.	2005	1456 (49%), 9-12y	Community, Cross-sec	Australia	PedsQL	Weight status[f]	No difference in emotional functioning scores by weight status.
Emotional Regulation Studies (n = 14)							
Öz & Kivrak	2023	98 (62%), 11-17y	Clinical, Cross-sec	Türkiye	DERS	BMI centile[d]	OB children experience more emotional regulation difficulties than controls (p=.005).
Özyurt et al.	2022	130 (49%), 12-17y	Clinical, Cross-sec	Türkiye	DERS	BMI centile[a,d,l]	OB children experience more emotional regulation difficulties than controls (ηp2= .569, p= .000).
Pace et al.	2019	200 (51%), 6-12y	Clinical, Cross-sec	Italy	AIS, ERC	BMI centile[c]	Higher negative emotional lability (β = 7.20, p= .01) and lower emotional regulation (β= -1.30, p= .01) predict OB group membership. No association for negative emotionality.
Percinel et al.	2018	60 (87%), 11-18y	Clinical, Cross-sec	Türkiye	DERS	BMI centile[l]	OB children experience more emotional regulation difficulties than controls (t= 7.171, p<.001).
Yilmaz Kafali et al.	2021	123 (55%), 11-18y	Clinical, Cross-sec	Türkiye	DERS	BMI SDS[l]	No direct association between emotional regulation and BMI SDS.
Biggs et al.	2017	47 (48%), M= 11.8(2.57)y	Clinical, Cross-sec	Australia	BRIEF	BMI z-score[c]	No association between emotional control and BMI z-score.
Reinelt et al.	2020	158 (53%), M= 14.43(1.25)y	Clinical, Prosp *(4-6wk FU)*	Germany	ERQ	BMI[h]	Change in BMI from pre-post obesity treatment associated with emotional reappraisal (b= -.03, p= .003). No association with emotional suppression.
Shriver et al.	2019	153 (56%), 15y	Community, Prosp *(4y FU)*	USA	ERCA	BF%, BMI centile[a]	No association between emotional control at 15 and BF% at age 19.
Doom et al.	2023	2587 (FFCWS only) (49%), 5-15y	Community, Prosp *(10y FU)*	USA	CBCL	BMI z-score[a]	No direct association between emotional dysregulation at age 5 and BMI z-score at age 15.
Kelly et al.	2016	221 (52%), 8-17y	Community, Cross-sec	USA	CBCL	BMI z-score[a]	No association between emotional dysregulation and BMI z-score.
Rhee et al.	2021	92 (46%), 4-6y	Community, Cross-sec	USA	BRIEF	BMI centile[a]	No association between emotional control and BMI centile.
Çalişkan & Özyurt	2020	219 (49.3%), 4-6y	Community, Cross-sec	Türkiye	ERC	BMI centile[l]	Median scores on emotional regulation higher in OW/Ob children than NW children (p= .027).
Miller et al.	2016	133 (50%), M= 33.10(.06)mo	Community, Cross-sec	USA	Lab task	BMI z-score[a]	Better emotional self-regulation (non-food) associated with lower BMI z-score (β = -.22, p<.05).
Power et al.	2016	187 (48%), M= 57.40(5.20)mo	Community, Cross-sec	USA	DoG task	BMI z-score[a]	No association between emotional regulation and BMI z-score.
General Affect Studies- Negative (n = 36)							
Çolpan et al.	2018	96 (56%), 12-17y	Clinical, Cross-sec	Türkiye	SDQ	Weight status[a]	OB children experience more frequent emotional symptoms than controls (p<.001).
Hampel et al.	2021	313 (62%), 12-17y	Clinical, Cross-sec	Germany	SDQ	BMI SDS[h]	More emotional symptoms associated with higher BMI SDS (partial r= .19, p<.01).

Authors	Year	N (%F), age range/M(*SD*)[1]	Setting, Design	Country	Affect Measure	Adiposity Measure	Association between affect and adiposity
Blanco et al.	2020	100 (60%), 8-12y	Clinical, Cross-sec	Spain	STAIC	Weight status[f]	State and trait anxiety higher in OB children compared to NW (*State:* OR= 1.12, CI= 1.02-1.22, p<.01; *Trait:* OR=1.08, CI= 1.01-1.15, p<.01).
Sahin & Kirli	2021	115 (64%), 8-16y	Clinical, Cross-sec	Türkiye	STAI	BMI centile, weight status[d]	State and trait anxiety higher in OB children compared to controls (*State*: p= .004, *Trait*: p= .024). BMI centile associated with trait, not state anxiety (partial r= .280, p= .002).
Sepúlveda et al.	2020	235 (58%) 8-12y	Clinical, Cross-sec	Spain	STAIC	BMI z-score[m], Weight status[f]	Trait anxiety higher in OB and OW children compared to NW (p= .02). Higher trait anxiety associated with higher BMI z-score (partial r= .20, p= .01).
Topçu et al.	2016	367 (64%), 9-16y	Clinical, Cross-sec	Türkiye	STAIC	Weight status[a]	State and trait anxiety higher in OB children than controls (p<.001).
Vila et al.	1995	96 (100%), 13-19y	Clinical, Cross-sec	France	STAIC-T	BMI z-score[n]	No difference in anxiety between OB and Non-OB girls.
Selewski et al.	2013	138 (54%), 8-17y	Clinical, Cross-sec	USA	PROMIS	BMI centile[a]	BMI >99th centile associated with higher anger (β= 5.2, CI= 1.1-9.3, p<.05). No association for anxiety.
Donnchadha et al.	2023	6500 (51%) 9-13y	Community, Prosp *(4yr FU)*	Ireland	SDQ	Weight status[f]	No association between emotional symptoms at age 9, and weight status at age 13.
Rosenthal et al.	2015	644 (56%), 10-12y	Community, Prosp (*2y FU)*	USA	SDQ	BMI[c]	Increase in BMI from the age 10-12y to 12-14y associated with emotional symptoms at 12-14y (B= .15, p= .047).
Kubzansky et al.	2012	1528 (51%), 11-18y	Community, Prosp *(2yr FU)*	USA	STAI	Age-and-gender adjusted-BMI trajectory[c]	Greater trait anxiety at baseline associated with higher likelihood of OB trajectory class membership 1 and 2 years later (*1 year:* OR= 1.33, CI= 1.08-1.62; *2 years:* OR = 1.29, CI= 1.05-1.59).
Bjertnaes et al.	2020	3189 (51%), 15-16y	Community, Cross-sec	Norway	SDQ	BMI centile[f,o]	Emotional symptoms associated with higher BMI in girls only (β= .07, CI= .01-.13). No association in boys and girls combined.
Donkor et al.	2021	1088 (53%), M= 5.72 (.43)y	Community, Cross-sec	Norway	SDQ	BMI centile[f,o]	UW and OB children had greater emotional symptoms than NW or OW children (p= .011).
Drukker et al.	2009	1411 (50%), 5-14y	Community, Cross-sec	Netherlands	SDQ	Weight status[p]	No association between emotional symptoms and weight category at 5-6years or 13-14years.
Förster et al.	2023	2350 (49%), 4-18y	Community, Cross-sec	Germany	SDQ	BMI SDS[h]	Higher BMI SDS associated with greater emotional symptoms in 11-18y sample (b= .10, p= .04), but not 4-10y sample.
Kohlboeck et al.	2014	2827 (49%), 10y	Community, Cross-sec	Germany	SDQ	BMI tertiles[c]	No association between emotional symptoms and BMI tertile.
Mallan et al.	2017	194 (48%), 3.5-4y	Community, Cross-sec	Australia	SDQ	BMI z-score[d]	No direct association between emotional symptoms and BMI z-score.
Noonan & Fairclough	2019	6011 (51%), 7y	Community, Cross-sec	UK	SDQ	Weight status[f]	Emotional symptoms only associated with weight status in boys who are in second least active quartile (Beta= .08, CI= .03-.14, p<.01). No association in girls for any quartile.
Pitrou et al.	2010	1030 (51%), 6-11y	Community, Cross-sec	France	SDQ	Weight status[f]	No association between emotional symptoms and weight status.
Ren et al.	2018	3841 (50%), 11-16y	Community, Cross-sec	China	SDQ	Weight status[q]	No association between emotional symptoms and weight status.
Shinsugi et al.	2021	508 (62%), 5-10y	Community, Cross-sec	Sri Lanka	SDQ	BMI-for-age-z-score[d]	No association between emotional symptoms and BMI-for-age.
Byrne et al.	2023	651 (66%), 7-18y	Community, Cross-sec	USA	STAIC	BMI z-score[a], fat mass (kg)	Trait anxiety associated with higher adiposity (b= .02, p<.01), and mediates the association between racial identity and adiposity (ab= .03 CI= .01-.05)
Crewther et al.	2024	120 (0%), 14-19y	Community, Cross-sec	Poland	STAI	BMI[c]	No association between anxiety and BMI.
Grammer et al.	2018	257 (53%), 8-17y	Community, Cross-sec	USA	STAIC	BMI z-score[a], fat mass (kg)	No direct association between anxiety and BMI z-score.

Authors	Year	N (%F), age range/M(*SD*)[1]	Setting, Design	Country	Affect Measure	Adiposity Measure	Association between affect and adiposity
Hanć et al.	2014	575 (54%), 10-15y	Community, Cross-sec	Poland	STAIC	BMI z-score[d]	No association between anxiety and BMI z-score.
Hillman et al.	2010	198 (100%), 11-17y	Community, Cross-sec	USA	STAIC, STAI	BF%; BMI z-score[a]	Trait anxiety associated with higher BF% (B= .103, p<.05). No association with BMI z-score.
Steen et al.	1996	224 (51%), 15-16y	Community, Cross-sec	USA	STAI	BMI[c]	NW girls show greater anxiety than OB boys (t= -2.78, p<.01). No difference between OB and NW girls.
Aditya & Sekartini	2017	384 (48%), 6-12y	Community, Cross-sec	Indonesia	PSC-17 (yes/no- defined by >5 score)	Weight status[p]	No association between PSC internalising score and weight status.
Ward-Begnoche et al.	2011	53 (66%), “middle school” (NS)	Community, Cross-sec	USA	CDI	BMI centile[c]	No differences in negative mood by weight status.
Czepczor-Bernat et al.	2020	282 (53%), M= 12.23 (2.80)y	Community, Cross-sec	Poland	PANAS- N subscale	BMI centile[r]	No direct association between negative affect and BMI centile.
Ivarsson et al.	2006	405 (52%), M= 14.65 (1.35)y	Community, Cross-sec	Sweden	CDI	Deviance from ideal BMI[s]	Negative mood associated with lower BMI in girls (B= -.25, p = .027). No association in boys.
Johnson & Greene	1991	78 (0%) 14-16y	Community, Cross-sec	USA	STAXI	Weight (lbs)	Higher weight in adolescents with high levels of suppressed anger compared to moderate and low (F= 6.26, p<.01)
Li et al.	2007	3886 (49%), 9-10y	Community, Cross-sec	China	CDI	BMI centile[q]	No difference in negative mood by weight status.
Riahi et al.	2019	14,440 (49%) 7-18y	Community, Cross-sec	Iran	Single items ‘worry, ‘anxiety’, ‘depressed’	Weight status[d]	No difference in worry, anxiety, or depression by weight status.
Stival et al.	2022	6014 (49%), 8-15y	Community, Cross-sec	Italy	Single items, 'low', 'nervous' 'irritable', averaged for ‘psychological distress’	Weight status[d,f]	OB children more likely to feel psychological distress (OR= 2.44, CI= 1.12-5.27) and nervous (OR= 2.37, CI= 1.32- 4.26) than NW. Irritability and nervousness, but not feeling low, increases with BMI level (irritable p= .038, nervous p= .007).
Suchert et al.	2016	1011 (NS), M= 14.10 (.60)y	Community, Cross-sec	Germany	CES-D	BMI centile[h]	Depressed affect does not mediate relationship between BMI centile and educational attainment.
General Affect Studies- Positive (n = 6)							
Kelly et al.	2016	16936 (49%), 3y	Community, Prosp *(8y FU)*	UK	Happiness scale (6-item)	BMI trajectory[f]	"High" or "Moderate" increasing growth trajectories between 3-11years associated with lower happiness at age 11, compared to "Stable" trajectory (HI OR = 2.07, p<.01; MI OR = .66, p <.01). No difference between "Stable" and "Decreasing" trajectory groups.
van Grieken et al.	2013	2372 (50%), 5y	Community, Prosp *(2y FU)*	Netherlands	Single item- happiness	Weight status, trajectory[f]	No association between happiness at age 7 and weight category at age 5, or weight trajectory between 5-7 years.
Sutin et al.	2021	1539 (43%), 15y	Community, Prosp *(1y FU)*	China	OHQ	BMI[c]	No association between BMI at age 15 and happiness at age 16.
Giacomo et al.	2019	593 (NS), >18y	Community, Cross-sec	Italy	EPOCH Happiness	BMI[d]	No differences in happiness by weight status.
Min et al.	2017	370568 (47%), 12-18y	Community, Cross-sec	South Korea	Single-item happiness	Weight status[t]	Underweight adolescents happier than normal weight adolescents (OR= 1.05, CI= 1.02-1.08, p<.001).
Vaquero-Soliś et al.	2021	452 (43%), 12-15y	Community, Cross-sec	Spain	SHS	BMI[c]	No association between happiness and BMI.
Mixed Affectual Valence Studies (n = 10)							
Pan et al.	2018	72 (24%), 4-15y	Clinical, Cross-sec	China	PANAS	Weight status[u]	Children with ‘Intermediate’ degree of obesity score lower on PANAS than those with ‘Mild’ (p= .026).
Jeffery et al.	2014	208 (45%), 7y	Community, Prosp *(9y FU)*	UK	PANAS-C	BF%, BMI SDS[g]	No association between changes in fat% between 7-16 years and lower mood at 16.

Authors	Year	N (%F), age range/M(*SD*)[1]	Setting, design	Country	Affect Measure	Adiposity Measure	Association between affect and adiposity
Alves et al.	2021	64 (63%), 9-15y	Community, Cross-sec	USA	PANAS	Weight status[a]	No difference in positive or negative affect between children with OW/OB and NW children.
Gil-Madrona et al.	2019	786 (48%), 10-13y	Community, Cross-sec	Spain	PANAS	BMI[c]	Children with OB score lower on positive affect than children without (F= -1.543, p= .031). No differences for negative affect.
McCabe et al.	2005	412 (52%), 8-11y	Community, Prosp *(16mo FU)*	Australia	PANAS-C	Weight status[f]	OW children scored higher in negative affect (F(1,378)= 8.52, p< .01), and lower in positive affect (F(1,378)= 8.21, p< .01) than non-OW. No interaction between weight status and time.
Alexius et al.	2012	1048 (49%), 6-10y	Community, Cross-sec	Brazil	Visual Analogue Scale (1-7)	Weight status[f]	Higher prevalence of OW/OB amongst children reporting 'unfavourable' psychological wellbeing vs 'favourable' psychological wellbeing (PR= 1.52, CI= 1.02-2.28), p= .038).
Hallal et al.	2010	4426 (NS), M = 11(NS)y	Community, Cross-sec	Brazil	Visual Analogue Scale (1-7)- from 'very happy' to 'very sad'	Weight status[v]	No association between weight status and likelihood of rating self 'very happy' or 'very sad'.
Fonseca et al.	2009	6131 (51%), 11-15y	Community, Cross-sec	Portugal	Single items. Happiness; Frequency of nerves/ bad temper	Weight status[f]	OB and OW groups more likely to describe themselves as unhappy ($\chi 2$= 15.60, p<.05) than non-OW/OB counterparts. OB more likely to be nervous ($\chi 2$= 26.32, p<.005) and irritable/bad-tempered ($\chi 2$= 23.28, p<.005).
Vanaelst et al.	2014	355 (48%), 5-10y	Community, Cross-sec	Belgium	SDQ, single items for happiness, sadness, anxiety, anger	WHtR, BMI z-score[f]	Happiness associated with lower BMI z-score (β = -.120, p = .044) and WHtR (β= -.148, p= .014). No associations for sadness, anxiety, anger or SDQ score.
Zhao et al.	2019	1081 (48%), 10-12y	Community, Cross-sec	China	CES-D	Weight status[q]	Weight status associated with lack of positive affect (OR= 1.04, CI= 1.03-1.05, p<.001) and negative affect (OR=1.06, CI= 1.02-1.10, p<.01).

[1] Age at baseline for prospective studies.
[2] China, Taiwan, Japan, Thailand, Philippines
Abbreviations: AIS- Affect Intensity Scale[17]; BF%- Body fat percentage; BMI- Body mass index; BRIEF- Behavior Rating of Executive Function[18]; CBQ- Child Behaviour Questionnaire[1]; CBCL- Child Behaviour Checklist[2]; CCTI- Colorado Child Temperament Inventory[3]; CDI- Children's Depression Index[19]; CES-D- Centre for Epidemiological Studies Depression Scale[20]; CSBQ- Child Social Behaviour Questionnaire[4]; DERS- Difficulties in Emotion Regulation Scale[21]; DoG task- Delay of Gratification task[22]; EAS- Emotionality, Activity and Sociability Scale[5]; EPOCH- Engagement, Perseverance, Optimism, Connectedness and Happiness measure[23]; ERC- Emotional Regulation Checklist[6]; ERICA- Emotional Regulation Index for Children and Adolescents[7]; ERQ- Emotional Regulation Questionnaire[8]; FU- maximum follow up period; IBQ- Infant Behaviour Questionnaire[9]; LAB-TAB – Laboratory Temperament Assessment Battery[10]; NS- not specified; OB- Obesity; OHQ- Oxford Happiness Questionnaire[24]; OW- Overweight; PANAS- Positive and Negative Affect Schedule[25]; PCERA- Parent-Child Early Relational Assessment[11]; PedsQL- Paediatric Quality of Life[12]; PROMIS- Patient-Reported Outcomes Measurement Information System[26]; PSC-17- Paediatric Symptom Checklist[27]; SDS- Standard deviation score; SDQ- Strengths and Difficulties questionnaire[13]; SHS- Subjective Happiness Scale[28]; SOB- Severe Obesity; STAI- State/Trait Anxiety Inventory[14]; STAXI- State/Trait Anger Expression Inventory[15]; TBAQ- Toddler Behaviour Assessment Questionnaire[29]; UW- Underweight; WC- Waist circumference; WHtR- Waist-to-height-ratio; WfL- Weight-for-length; WfA- Weight-for-age;
[a] Centre for Disease Control (CDC, 2000) Growth Reference Charts
[b] Child Growth Foundation 1996S
[c] None/Not Specified
[d] World Health Organisation (WHO) 2007
[e] National Institutes of Health 1991
[f] International Obesity Task Force (IOTF)
[g] UK Growth Reference 1990
[h] German population growth reference 2002
[i] World Health Organisation (WHO) Infant Growth Charts 2006
[j] Dutch population growth reference 2011
[k] Mix of national growth data from each country
[l] Turkish population growth reference 2006
[m] Spanish population growth reference 2000

[n] French growth reference 1991
[o] Norwegian population growth reference 2006
[p] National Centre Health statistics
[q] Working group for Obesity in China (WGOC)

[r] Polish population growth reference
[s] Swedish population growth reference 1994
[t] Korean population growth reference 2007
[u] Chinese population growth reference 2002
[v] World Health Organisation (WHO) 1995

Reference papers for included affect measures (Tables S1 and S2)

1 M. K. Rothbart, S. A. Ahadi, K. L. Hershey and P. Fisher, “Investigations of Temperament at Three to Seven Years: The Children’s Behavior Questionnaire,” *Child Development* 72, no. 5 (2001): 1394-1408, https://doi.org/10.1111/1467-8624.00355.

2 T. M. Achenbach, and T. M. Ruffle, “The Child Behavior Checklist and related forms for assessing behavioral/emotional problems and competencies,” *Pediatrics in Review* 21, no. 8 (2000): 265-71. https://doi.org/10.1542/pir.21-8-2652.

3 D. C. Rowe and R. Plomin, “Temperament in Early Childhood,” *Journal of Personality Assessment* 41, no. 2 (1977): 150-156, https://doi.org/10.1207/s15327752jpa4102_5.

4 C. A. Hartman, E. Luteijn, M. Serra and R. Minderaa, “Refinement of the Children’s Social Behavior Questionnaire (CSBQ): An Instrument That Describes the Diverse Problems Seen in Milder Forms of PDD,” Journal of Autism and Developmental Disorders 36, no. 3 (2006): 325-342, https://doi.org/10.1007/s10803-005-0072-z.

5 A. H. Buss and R. Plomin, “Temperament: Early Developing Personality Traits,” Hillsdale, NJ: Lawrence Erlbaum.

6 A. Shields and D. Cicchetti, “Emotion Regulation Among School-Aged Children: The Development and Validation of a New Criterion Q-sort Scale,”, *Developmental Psychology* 33 (1997): 906-916.

7 S. T. MacDermott, E. Gullone, J. S. Allen, et al., “The Emotion Regulation Index for Children and Adolescents (ERICA): A Psychometric Investigation,” *Journal of Psychopathology and Behavioral Assessment* 32 (2010): 301-314, https://doi.org/10.1007/s10862-009-9154-0.

8 J. J. Gross and O. P John, “Individual Differences in Two Emotion Regulation Processes: Implications for Affect, Relationships and Well-being”, *Journal of Personality and Social Psychology* 85, no. 2 (2003): 348-362, https://doi.org/10.1037/0022-3514.85.2.348.

9 M. K. Rothbart, “Measurement of Temperament in Infancy”, *Child Development* 52, no. 2 (1981): 569-578, https://doi.org/10.2307/1129176.

10 W. B. Carey and S. C McDevitt, “Revision of the Infant Temperament Questionnaire”, *Pediatrics* 61, no 5. (1978): 735-739.

11 H. H. Goldsmith and M. K. Rothbart, *The Laboratory Temperament Assessment Battery (LAB-TAB): Prelocomotor Version 3.0, Technical Manual* (Department of Psychology, University of Wisconsin, 1996).

12 R. Clark, *The Parent-Child Early Relational Assessment. Instrument and Manual* (Department of Psychiatry, University of Wisconsin Medical School, 1985).

13 J. W. Varni, M. Seid and P. S. Kurtin, “PedsQL™ 4.0: Reliability and validity of the pediatric quality of life inventory™ version 4.0 generic core scales in healthy and patient populations,” *Medical Care* 39, no. 8 (2001):800-812. https://doi.org/10.1097/00005650-200108000-00006.

14 R. Goodman, “The strengths and difficulties questionnaire: a research note,” *Journal of Child Psychology and Psychiatry* 38, no. 5 (1997): 581-586. https://doi.org/10.1111/j.1469-7610.1997.tb01545.x.

15 C. D. Spielberger, C. D. Edwards, J. Montouri and R. Lushene, *State-Trait Anxiety Inventory for Children* (Consulting Psychologists Press, 1973), https://doi.org/10.1037/t06497-000.

16 C. D. Spielberger, *Manual for the State-Trait Anger Expression Inventory (STAXI)* (Psychological Assessment Resources, 1988).

17 R. J. Larsen and E. Diener, “Affect Intensity as an Individual Difference Characteristic: A Review,” *Journal of Research in Personality* 21 (1987): 1-39, https://doi.org/10.1016/0092-6566(87)90023-7.

18 G. A. Gioia, P. K. Isquith, P. D. Retzlaff and K. A Espy, “Confirmatory Factor Analysis of the Behavior Rating Inventroy of Executive Function (BRIEF) in a Clinical Sample,” *Child Neuropsychology* 8, no. 4 (2002): 249:257, https://doi.org/10.1076/chin.8.4.249.13513.

19 M. Kovacs, *The Children’s Depression Inventory (CDI) Manual* (Multi-Health Systems, 1992),

20 L. S. Radloff, “The CES-D Scale: A Self-Report Depression Scale for Research in the General Population,” *Applied Psychological Measurement* 1, no. 3 (1977):385-401, https://doi.org/10.1177/014662167700100306.

21 K. L Gratz and L. Roemer, “Multidimensional Assessment of Emotion Regulation and Dysregulation: Development, Factor Structure and Initial Validation of the Difficulties in Emotion Regulation Scale,” *Journal of Psychopathology and Behavioral Assessment* 26, no. 1 (2004):41-54: https://doi.org/10.1023/b:joba.0000007455.08539.94.

22 H. N. Mischel and E. B. Ebbesen, “Attention in Delay of Gratification,” *Journal of Personality and Social Psychology* 16 (1970): 329-337, https://doi.org/10.1037/h0032198.

23 M. L. Kern, L. Benson, E. A Steinberg and L. Steinberg, “The EPOCH Measure of Adolescent Well-Being,” *Psychological Assessment* 28, no. 5 (2016): 586-597, https://doi.org/10.1037/pas0000201.

24 P. Hills and M. Argyle, “The Oxford Happiness Questionnaire: a Compact Scale for the Measurement of Psychological Well-Being,” *Personality and Individual Differences* 33 (2022): 1072-1082, https://doi.org/10.106/S0191-8869(01)00213-6.

25 J. Laurent, S. J. Catanzaro, T. E. Joiner et al., “A Measure of Positive and Negative Affect for Children: Scale Development and Preliminary Validation,” *Psychological Assessment* 11, no. 3 (1999): 326-338, https://doi.org/10.1037/1040-3590.11.3.326.

26 D. E. Irwin, B. Stucky, M. M. Langer, et al., “An Item Response Analysis of the Pediatric PROMIS Anxiety and Depressive Symptoms Scales,” *Quality of Life Research* 19, no. 4 (2010): 595-607, https://doi.org/10.1007/s11136-010-96193.

27 W. Gardner, M. Murphy, G. Childs, K. Kelleher and R. Sturner, “The PSC-17: a Brief Pediatric Symptom Checklist with Psychosocial Problem Subscales. A Report from PROS and ASPN,” *Ambulatory Child Health* 5, no. 3 (1999): 225-236.

28 S. Lyubomisky and H. S. Lepper, “A Measure of Subjective Happiness: Preliminary Reliability and Construct Validation,” *Social Indicators Research* 46, no. 2 (1999): 137-155, https://doi.org/10.1032/A:1006824100041.

29 H. H. Goldsmith, “Studying Temperament via Construction of the Toddler Behavior Assessment Questionnaire,” *Child Development* 67, no. 1 (1996): 218-235, https://doi.org/10/2307/1131697.

File S5: Summary of studies examining associations using simple correlations

In total, 39 studies tested simple correlations between affect and adiposity. These largely focused on negative affect (17/39)[1-17] followed by emotional functioning (6/39),[18-23] emotional regulation (6/39)[24-29] and mixed valence affect (6/39).[30-35] Two studies explored a combination of negative affect and another aspect of affect (i.e. functioning and regulation),[36,37] and two focused on positive affect only.[38,39] Twenty-four of the 38 studies found null associations, including all studies examining both positive and negative affect (5/5). Of the 15 studies that did find an association, higher adiposity was associated with lower emotional functioning (3/15),[18,33,36] poorer emotional regulation (3/15),[26,29,37] and higher negative affect (8/15). [2,5,8,10,13,14,17] One study reported 'low to moderate' correlations between positive and negative affect and BMI percentile, but these are not quantified.[32] One study found higher adiposity was associated with more positive affect, but for girls only (1/15).[39] Three studies found mixed associations, whereby lower emotional functioning, but not negative mood was associated with higher adiposity (1/15)[36]; poorer emotional regulation, but not negative affect was associated with higher adiposity (1/15),[37] and that higher adiposity was only associated with poorer scores in certain elements of emotional regulation, but not with others (1/15).[26]

References (File S5)

1 C. K. Ewart, G. J. Elder and J. M. Smyth, "How Neighborhood Disorder Increases Blood Pressure in Youth: Agonistic Striving and Subordination," *Journal of Behavioural Medicine* 37, no.1 (2014):113-26, https://doi.org/10.1007/s10865-012-9467-4.

2 L. Sim, and J. Zeman, "The Contribution of Emotion Regulation To Body Dissatisfaction and Disordered Eating in Early Adolescent Girls," *Journal of Youth and Adolescence* 35, no. 2 (2006): 207-216, https://doi.org/10.1007/s10964-005-9003-8.

3 P. Isnard, G. Michel, M. L. Frelut, *et al., "*Binge Eating and Psychopathology In Severely Obese Adolescents," *International Journal of Eating Disorders* 34 (2003): 235-243, https://doi.org/10.1002/eat.10178.

4 P. Isnard, L. Quantin, S. Cortese, *et al., "*Bulimic Behaviours and Psychopathology in Obese Adolescents and in Their Parents". *International Journal of Pediatric Obesity* 5 (2010): 474-482, https://doi.org/10.3109/17477160903571987.

5 A. G. Rubin, N. A. Schvey, L. M. Shank*, et al,. "*Associations Between Weight-Based Teasing and D isordered Eating Behaviors Among Youth,". *Eating Behaviors* 41 (2021):101504, https://doi.org/10.1016/j.eatbeh.2021.101504.

6 S. Solomon, L. M. Shank, J. M., Lavender, et al. "The Relationship Between Anxiety, Coping, and Disordered-Eating Attitudes in Adolescent Military-Dependents At High-Risk for Excess Weight Gain," Military Psychology 35, no. 2 (2023): 95-106, https://doi.org/10.1080/08995605.2022.2083448.

7 D. Young-Hyman, M. Tanofsky-Kraff, S. Z. Yanovski*, et al., "*Psychological Status and Weight-Related Distress in Overweight or At-Risk-for-Overweight Children," *Obesity (Silver Spring)* 14, no. 12 (2006):2249-2258, https://doi.org/10.1038/oby.2006.264.

8 A. Ternouth, D. Collier, and B. Maughan, "Childhood Emotional Problems and Self-Perceptions Predict Weight Gain in a Longitudinal Regression Model," BMC *Medicine* 7, no. 1 *(*2009): 46, https://doi.org/10.1186/1741-7015-7-46.

9 C. Forrester-Knauss, S. Perren, and F. D. Alsaker, "Does Body Mass Index in Childhood Predict Restraint Eating in Early Adolescence?," *Appetite* 59, no. 3 (2012): 921-926, https://doi.org/10.1016/j.appet.2012.08.026.

10 J. Plumed, N. Gimeno, M. Barberá, *et al., "*Teasing As A Risk Factor For Abnormal Eating Behaviours: A Prospective Study in an Adolescent Population,". *Revisita de Psiquiatria Salud Mental (English Edition)* 12, no. 1 (2019):17-27, https://doi.org/10.1016/j.rpsmen.2017.06.002.

11 H. Chen, and T. Jackson, "Predictors of Changes in Body Image Concerns of Chinese Adolescents," *Journal of* Adolescence 32, no.4 (2009):977- 994, https://doi.org/10.1016/j.adolescence.2008.08.002.

12 T. A. Wadden, G. Brown, G. D. Foster, and J. R Linowitz, "Salience of Weight-Related Worries in Adolescent Males and Females," *International Journal of Eating Disorders* 10, no.4 (1991):407-414, https://doi.org/10.1002/1098-108x(199107)10:4<407::aid-eat2260100405>3.0.co;2-v.

13 B. K., Albayrak and Y. Kutlu, "The Determination of Blood Pressure, Anger Expression and Body Mass Index in Adolescents in Turkey: A Pilot Study," *Collegium Antropologicum.* 36, no.1 (2012):87-92.

14 E. H. Johnson, "Interrelationships Between Psychological Factors, Overweight, and Blood Pressure in Adolescents," *Journal*

of Adolescent Health Care 11, no.4 (1990):310-318, https://doi.org/10.1016/0197-0070(90)90041-Y.

15 M. K. Hupp, P. C. Papathakis, S. Phelan, and A. K. Ventura, "Associations Between Mothers' Use of Food To Soothe, Feeding Mode, and Infant Weight During Early Infancy," *Appetite* 168(2022):105736, https://doi.org/10.1016/j.appet.2021.105736.

16 J. L. Suisman, J. D. Slane, S. A. Burt, and K. L. Klump, "Negative Affect As A Mediator of The Relationship Between Weight-Based Teasing and Binge Eating in Adolescent Girls,". *Eating Behaviors* 9, no.4 (2008):493-96, https://doi.org/10.1016/j.eatbeh.2008.04.001.

17 K. Presnell, S. K. Bearman, and E. Stice, "Risk Factors For Body Dissatisfaction in Adolescent Boys and Girls: A Prospective Study," *International Journal of Eating Disorders* 36, no. 4 (2004): 389-401, https://doi.org/10.1002/eat.20045.

18 O. Pinhas-Hamiel, S. Singer, N. Pilpel, A. Fradkin, D. Modan, B. Reichman, "Health-Related Quality of Life Among Children and Adolescents: Associations With Obesity," *International Journal of Obesity (Lond)* 30, no.2 (2006): 267-272, https://doi.org/10.1038/sj.ijo.0803107.

19 D. Özalp Kizilay, S. Yalin Sapmaz, S. Şen, Y. Özkan, B. C. Özyurt, B. Ersoy, "Mental Health of Both Child And Parents Plays A Larger Role i n The Health-Related Quality of Life of Obese and Overweight Children," *Journal of Pediatric Endocrinology and Metabolism*. 32, no.12 (2019):1359-1367, https://doi.org/10.1515/jpem-2019-0401.

20 N. R. Kelly, S. E. Mazzeo, R. K Evans, *et al., "*Physical Activity, Fitness and Psychosocial Functioning of Obese Adolescents," *Mental Health and Physical Activity* 4, no.1 (2011):31-37, https://doi.org/10.1016/j.mhpa.2010.11.001.

21 S. Gunawardana, C. B. Gunasinghe, M. S. Harshani, and S. N. Seneviratne, "Physical and Psychosocial Quality of Life in Children With Overweight and Obesity From Sri Lanka," *BMC Public Health* 21, no.1 (2021):86, https://doi.org/10.1186/s12889-020-10104-w.

22 V. Guardabassi, A. Mirisola, and C. Tomasetto, "How Is Weight Stigma Related to Children's Health-Related Quality Of Life? A Model Comparison Approach," *Quality of Life Research* 27, no.1 (2018): 173-183, https://doi.org/10.1007/s11136-017-1701-7.

23 C. Meade, R. Martin, A. McCrann*, et al., "*Prader-Willi Syndrome in Children: Quality Of Life And Caregiver Burden," *Acta Paediatrica* 110, no. 5 (2021): 1665-1670, https://doi.org/10.1111/apa.15738.

24 T. Mohorić, A. Pokrajac-Bulian, P. Anić, M. Kukić, and P. Mohović, "Emotion Regulation, Perfectionism, and Eating Disorder Symptoms in Adolescents: The Mediating Role of Cognitive Eating Patterns," *Current Psychology* 42, no. 32 (2023): 28505-16, https://doi.org/10.1007/s12144-022-03824-2.

25 F. Laghi, D. Bianchi, S. Pompili, A. Lonigro, and R. Baiocco, "Metacognition, Emotional Functioning and Binge Eating in Adolescence: The Moderation Role of Need to Control Thoughts," *Eating and Weight Disorders* 23, no.6 (2018): 861-869, https://doi.org/10.1007/s40519-018-0603-1.

26 J. Vandewalle, E. Moens, and C., Braet, "Comprehending Emotional Eating in Obese Youngsters: The Role of Parental Rejection and Emotion Regulation," *International Journal of Obesity (London)* 38, no. 4 (2014): 525-530, https://doi.org/10.1038/ijo.2013.233.

27 S. E Domoff, E. Q. Sutherland, S. Yokum, and A. N. Gearhardt, "Adolescents' Addictive Phone Use: Associations With Eating Behaviors and Adiposity," *International Journal of Environmental Research and Public Health* 17, no.8 (2020): 2861. https://doi.org/10.3390/ijerph17082861.

28 M. J. Gouveia, M. C. Canavarro, and H. Moreira, "Associations Between Mindfulness, Self-Compassion, Difficulties in Emotion Regulation, and Emotional Eating Among Adolescents With Overweight/Obesity," *Journal of Children and Family Studies* 28, no.1 (2019): 273-85, https://doi.org.10.1007/s10826-018-1239-5.

29 D. Yeum, D. Gilbert-Diamond, T. D. Masterson, *et al., "*Associations Between Behavioral Self- Regulation and External Food Cue Responsiveness (EFCR) in Preschool-Age Children and Evidence of Modification by Parenting Style," *Appetite* 188(2023): 106637, https://doi.org/10.1016/j.appet.2023.106637.

30 S. Byrne, and D. S. Kirschenbaum, "Helping Young Weight Controllers Develop Healthy Obsessions: Preliminary Test of The Healthy Obsession Model," *Clinical Obesity* 1, no 2-3 (2011): 85-91, https://doi.org/10.1111/j.1758-8111.2011.00018.x.

31 J. J. Chiang, J. E. Bower, D. M. Almeida, M. R. Irwin, T. E. Seeman, A. J Fuligni, "Socioeconomic Status, Daily Affective and Social Experiences, and Inflammation During Adolescence,". *Psychosomatic Medicine* 77, no.3 (2015): 256-266, https://doi.org/10.1097/psy.0000000000000160.

32 M. J. Gilliland, M. Windle, J. A. Grunbaum*, et al., "*Body Image and Children's Mental Health Related Behaviors: Results from the Healthy Passages Study," *Journal of Pediatric Psychology* 32, no.1 (2007): 30-41, https://doi.org/10.1093/jpepsy/jsl008.

33 S. M. Manasse, A. A. Haedt-Matt, K. E. Smith, *et al., "*The Moderating Role of Sleep Duration on Momentary Relations Between Negative Affect and Loss-Of-Control Eating in Children and Adolescents," *European Eating Disorders Review* 30, no.6 (2022): 815-822, https://doi.org/10.1002/erv.2908.

34 T. B. Mitchell, and R. G. Steele, "The Effect of Body Mass Index, Negative Affect, and Disordered Eating on Health-Related Quality of Life in Preadolescent Youth," *Journal Pediatric Psychology* 41, no.7 (2016): 768-776, https://doi.org/10.1093/jpepsy/jsv163.

35 D. J. Korczak, S. Madigan, M. Colasanto, *et al., "*The Longitudinal Association Between Temperament and Physical Activity in Young Children," *Preventative Medicine* 111(2018): 342-347, https://doi.org/10.1016/j.ypmed.2017.11.021.

36 B. N. Whitaker, P. L. Fisher, S. Jambhekar*, et al., "*Impact of Degree of Obesity on Sleep, Quality of Life, and Depression in Youth," *Journal of Pediatric Health Care* 32, no.2 (2018): e37-44, https://doi.org/10.1016/j.pedhc.2017.09.008.

37 E. L. Osborne, B. Ainsworth, P. Chadwick, and M. J. Atkinson, "The Role of Emotion Regulation in the Relationship Between Mindfulness and Risk Factors for Disordered Eating: A Longitudinal Mediation Analysis,". *International Journal of Eating Disorders* 56, no.2 (2023): 458-463, https://doi.org/10.1002/eat.23849.

38 A. L Duckworth, E. Tsukayama, and A. B. Geier, "Self-Controlled Children Stay Leaner in The Transition To Adolescence," *Appetite*. 54, no.2 (2010) :304-308, https://doi.org/10.1016/j.appet.2009.11.016.

39 M. P McCabe, L. A Ricciardelli, K. Holt, "Body Image, Strategies to Change Muscles and Weight, and Puberty: Do They Impact on Positive a nd Negative Affect Among Adolescent Boys and Girls?" *Eating Behaviors* 2, no.2 (2001): 129-149, https://doi.org/10.1016/S1471-0153(01)00025-3.
